# Mitochondria are secreted in extracellular vesicles when lysosomal function is impaired

**DOI:** 10.1038/s41467-023-40680-5

**Published:** 2023-08-18

**Authors:** Wenjing Liang, Shakti Sagar, Rishith Ravindran, Rita H. Najor, Justin M. Quiles, Liguo Chi, Rachel Y. Diao, Benjamin P. Woodall, Leonardo J. Leon, Erika Zumaya, Jason Duran, David M. Cauvi, Antonio De Maio, Eric D. Adler, Åsa B. Gustafsson

**Affiliations:** 1 Skaggs School of Pharmacy and Pharmaceutical Sciences, University of California San Diego, La Jolla, CA USA; 2 Department of Medicine, University of California San Diego, La Jolla, CA USA; 3 Department of Surgery, University of California San Diego, La Jolla, CA USA; 4 Department of Pharmacology, University of California San Diego, La Jolla, CA USA

**Keywords:** Mitochondria, Cardiovascular biology, Secretion

## Abstract

Mitochondrial quality control is critical for cardiac homeostasis as these organelles are responsible for generating most of the energy needed to sustain contraction. Dysfunctional mitochondria are normally degraded via intracellular degradation pathways that converge on the lysosome. Here, we identified an alternative mechanism to eliminate mitochondria when lysosomal function is compromised. We show that lysosomal inhibition leads to increased secretion of mitochondria in large extracellular vesicles (EVs). The EVs are produced in multivesicular bodies, and their release is independent of autophagy. Deletion of the small GTPase Rab7 in cells or adult mouse heart leads to increased secretion of EVs containing ubiquitinated cargos, including intact mitochondria. The secreted EVs are captured by macrophages without activating inflammation. Hearts from aged mice or Danon disease patients have increased levels of secreted EVs containing mitochondria indicating activation of vesicular release during cardiac pathophysiology. Overall, these findings establish that mitochondria are eliminated in large EVs through the endosomal pathway when lysosomal degradation is inhibited.

## Abstract (web summary)

Mitochondrial quality control is critical for cellular homeostasis and survival. Here, the authors identify that defective mitochondria can be eliminated via secretion in large extracellular vesicles when internal lysosomal degradation is compromised.

## Introduction

Cardiac myocyte contraction requires high levels of ATP which is supplied by mitochondrial oxidative phosphorylation. However, dysfunctional mitochondria generate excessive reactive oxygen species (ROS) that can cause damage to cellular components, and these organelles can also directly activate cell death pathways^1^. Thus, efficient removal of aberrant mitochondria is crucial to maintain cellular homeostasis and cardiac function. To prevent unnecessary death, cells have evolved various mechanisms involved in repairing or removing dysfunctional mitochondria^2^. These quality control pathways are particularly important in terminally differentiated cardiac myocytes, which are unable to dilute cellular damage through cell division and cannot be easily regenerated. Autophagy is the primary pathway involved in clearing dysfunctional mitochondria in the heart^3–5^ and involves the sequestration of a mitochondrion by an autophagosome which then delivers the cargo to a lysosome^2^. Mitochondria can also be engulfed by early endosomes^6,7^, or directly taken up into lysosomes^8^. Although multiple mechanisms of mitochondrial degradation exist in cells, these pathways all converge at the lysosome which is ultimately responsible for the final breakdown of protein aggregates and organelles^9^. Whether alternative mitochondrial quality control pathways exist when lysosomal function is compromised or overwhelmed is currently unclear.

Cells are known to release vesicles from different origins that range from 0.05–1 μm in diameter into the extracellular space^10^. Extracellular vesicles (EVs) are released after fusion of late endosomes (LE)/multivesicular bodies (MVB) with the plasma membrane or via budding of the plasma membrane. Studies have reported that these vesicles participate in cell communication by delivering nucleic acids, proteins, and lipids to recipient cells^10,11^. The release of EVs is increased in various diseases or during stress. For instance, there is increased release of EVs from the heart during a myocardial infarction^12–14^. Secretion of EVs by skeletal muscle is also increased during exercise in both rodents and humans^15^. The EVs have been reported to promote regeneration of injured skeletal muscle tissue^16^. However, there is also emerging evidence that EVs might function as an alternative pathway of cellular quality control. EVs containing β-amyloid and α-synuclein have been detected in patients with Alzheimer’s disease and Parkinson’s disease, respectively^17,18^. Cells have also been reported to release large (3.5–4 μm) subcellular structures known as exophers which are much larger than traditional EVs^19,20^. Intriguingly, several studies have identified mitochondrial proteins as EV cargo^20–23^, but their function and mechanism of release are still poorly understood. Formation and trafficking of endosomal vesicles containing EVs are regulated by Rab GTPases^10^. Rab7 is present on the LE/MVB and is required for the fusion between the LE/MVB and the lysosome^24^. It is also involved in regulating autophagosome-lysosome fusion^25,26^, indicating crosstalk between the two internal degradation pathways. Whether Rab7 plays a role in regulating fusion between the LE/MVB with the plasma membrane to release EVs into the extracellular space is still unclear.

Danon disease is caused by loss-of-function mutations in the lysosome-associated membrane protein 2 (*LAMP2)* gene, which leads to impaired autophagic degradation and development of cardiomyopathy^27,28^. Interestingly, these patients are often asymptomatic in early childhood, suggesting that alternative mechanisms compensate for defective autophagic-lysosomal degradation. Here, we describe a different mechanism of mitochondrial clearance in cells that involves secretion of mitochondria in large EVs when Rab7 is deleted or lysosomal function is compromised. This mechanism might function as an alternative cellular quality control pathway when lysosomal function is compromised to protect cells against accumulation of harmful cargo that is destined for degradation.

## Results

### Inhibiting lysosomal acidification leads to increased release of large and small extracellular vesicles

**Fig. 1:**
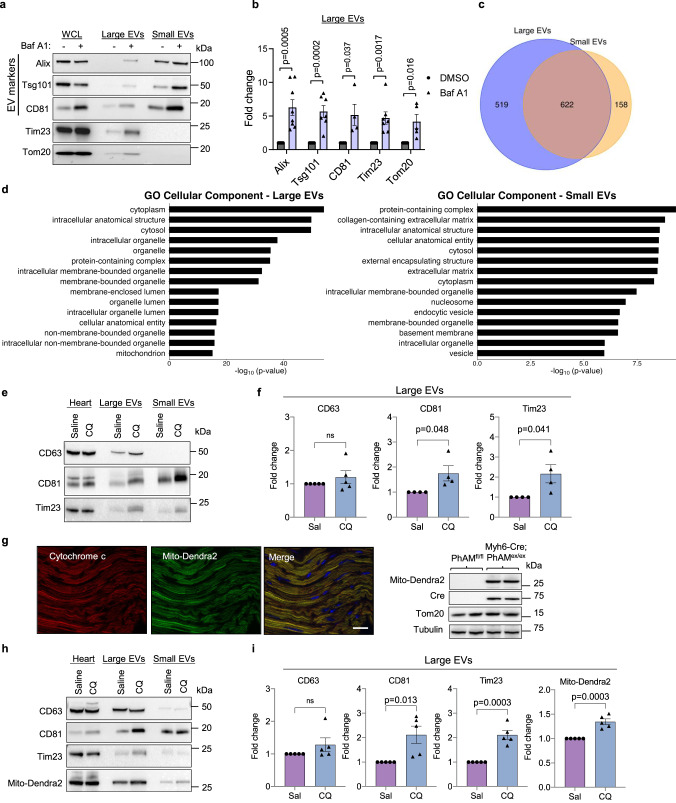
Inhibiting lysosomal acidification leads to increased release of large and small extracellular vesicles (EVs) from cells and hearts. **a** Representative Western blots of whole cell lysate (WCL) and extracellular vesicle fractions for EV marker proteins Alix, Tsg101, CD81 and the mitochondrial proteins Tim23 and Tom20. The EVs were obtained by differential centrifugation of conditioned media from mouse embryonic fibroblasts collected over 48 h after treatment with vehicle or Bafilomycin (Baf A1, 5 nM). **b** Quantification of proteins in large EVs (Alix *n* = 8; Tsg101, Tim23 *n* = 7; Tom20 *n* = 5; CD81 *n* = 4 biologically independent experiments). **c** Venn diagram showing overlap of proteins detected in large and small EVs. **d** Gene ontology (GO) analysis of the unique 519 proteins identified in large EV fractions with the top 15 terms for cellular component plotted according to –log10False Discovery Rate (FDR). **e** Representative Western blot analysis of EV proteins CD63, CD81 and the mitochondrial protein Tim23 in heart lysates and EV fractions isolated from heart tissue. **f** Quantification of proteins in large EVs fraction (CD63 *n* = 5; DC81, Tim23 *n* = 4 biologically independent experiments). **g** Photo-activatable mitochondria (*PhAM*^floxed^) mice were crossed with *Myh6*-Cre transgenic mice to generate a mouse line (*PhAM*^*excised*^) with cardiac specific expression of Mito-Dendra2. Expression of Mito-Dendra2 (green) and Cre was confirmed by imaging of frozen heart sections (representative of *n* = 2 biologically independent samples) and by Western blotting of heart lysates (*n* = 3 biologically independent samples). Heart sections were stained with anti-Cytochrome c (red) to label mitochondria. Scale bar = 20 μm. **h** Representative Western blot analysis of CD63, CD81, Tim23, and Mito-Dendra2 in heart lysates and EV fractions isolated from heart tissue. **i** Quantification of proteins in large EVs (*n* = 5 biologically independent experiments). Data are mean ± SEM. ns = not significant. *P* values shown are by two-sided Student’s *t*-test. Source data are provided as a Source Data file.

To investigate the relationship between lysosomal impairment and EV secretion, we treated mouse embryonic fibroblasts (MEFs) with the vacuolar H+ ATPase (V-ATPase) inhibitor Bafilomycin A1 (Baf A1) and examined EV release from these cells. Large and small EVs were collected from the conditioned medium using differential centrifugation or affinity purification protocols^29^ (Supplementary Fig. 1a). Western blotting of EV fractions isolated from conditioned media showed that both large and small EV fractions were positive for Alix, Tsg101, and CD81, which are established markers of EVs derived from the MVBs^10^. These EV markers were significantly increased after Baf A1 treatment (Fig. 1a, b and Supplementary Fig. 1b, c), indicating increased secretion of EVs during inhibition of lysosomal acidification. Interestingly, the large EV fraction, but not small EV fraction, was also positive for the outer and inner mitochondrial membrane proteins Tom20, Tim23 and MTCO1 and treatment with Baf A1 significantly increased mitochondrial protein levels in large EVs (Fig. 1a, b and Supplementary Fig. 1c). Note that the extended treatment (48 h) with BafA1 at nanomolar concentrations led to increased death in about ~20% of the cells (Supplementary Fig. 1d), suggesting that chronic abrogation of lysosomal acidification is cytotoxic. In addition, mitochondrial depolarization with FCCP treatment, a potent inducer of mitophagy^30^, was also associated with increased secretion of large EVs containing mitochondria in MEFs (Supplementary Fig. 1e). In contrast, FCCP treatment led to a significant reduction in the secretion of small EVs.

Next, we performed proteomics analysis of large and small EVs secreted by MEFs under baseline conditions to compare their composition and cargo. Among the proteins identified in small EVs, 80% (622/780) of these proteins were also present in large EVs (Fig. 1c). We also confirmed that proteins identified in large EVs overlapped with proteins in the exosomal and EV molecular databases Vesiclepedia (http://microvesicles.org/index.html) and Exocarta (www.exocarta.org) (Supplementary Fig. 1f). Furthermore, Gene Ontology (GO) enrichment analysis of the 519 proteins exclusively found in the large EVs showed an enrichment of membrane bound organelles including mitochondria (Fig. 1d and Supplementary Data 1). In contrast, the small EVs showed an enrichment in protein-containing complex, cytosol and cytoplasm (Fig. 1d). Overall, these findings suggest that disrupting lysosomal acidification leads to increased secretion of large and small EVs from cells and that large EVs may contain intact mitochondria.

To investigate if disrupting lysosomal function enhances the release of EVs in vivo, we administered chloroquine (CQ), a lysosomotropic drug that increases lysosomal pH, to mice and investigated changes in EV content within the extracellular space of heart tissue and in plasma isolated 24 h after administration (Supplementary Fig. 2a). While we observed no increase in circulating EVs in plasma after CQ administration (Supplementary Fig. 2b), we found that CQ treatment led to increased levels of large EVs in cardiac tissue (Fig. 1e, f and Supplementary Fig. 2c). Similar to what we observed in MEFs, levels of the mitochondrial protein Tim23 increased in the large EV fraction with CQ treatment. To investigate whether the mitochondria in large EVs originated from cardiac myocytes, we generated mice with cardiac-specific expression of the Dendra2 fluorescent protein in mitochondria (mito-Dendra2) by breeding photo-activatable mitochondria (PhAM^f/f^) and Myh6-Cre mice (Fig. 1g). Large EV fractions isolated from hearts of mito-Dendra2 mice using differential centrifugation were positive for both Tim23 and mito-Dendra2 (Fig. 1h, i) suggesting that cardiac myocytes are the primary source of mitochondria containing EVs released into the extracellular space. To further validate our findings, we isolated EVs from cardiac tissue using affinity purification with bead conjugated antibodies against EV cell surface markers. These fractions were also positive for Tim23 and mito-Dendra2, and were significantly increased in mice treated with CQ (Supplementary Fig. 2d). Together, these results suggest that mitochondria can be released in large EVs originating from the endosomal system when lysosomal function is compromised in the mouse heart.

### Rab7-deficiency leads to increased secretion of large and small EVs

**Fig. 2:**
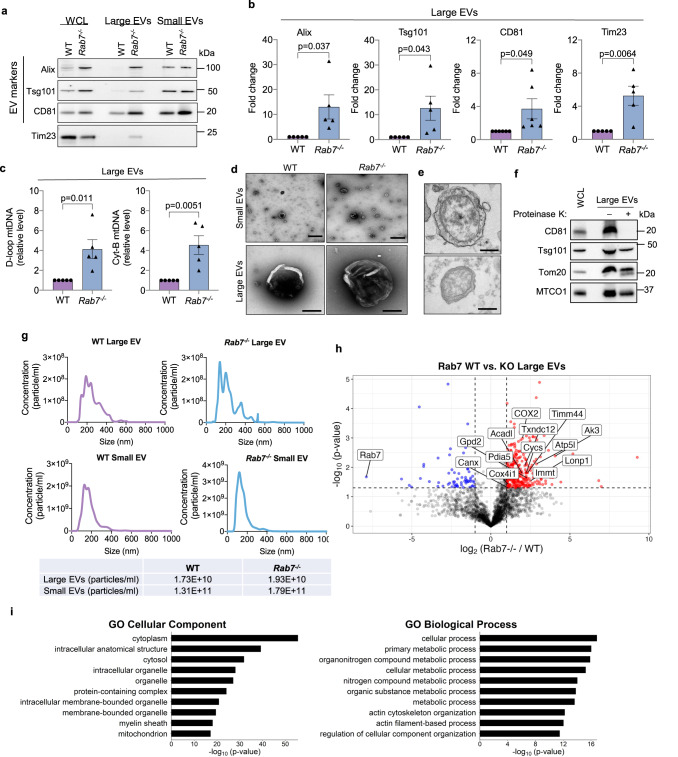
Rab7-deficient cells have increased EV secretion at baseline. **a** Representative Western blots of Alix, Tsg101, CD81, and Tim23 in total cell lysates and EV preparations from WT and *Rab7*^*−/*−^ MEFs. **b** Quantification of proteins in large EV fractions (Alix, Tsg101, Tim23 *n* = 5; CD81 *n* = 6 biologically independent experiments). **c** Mitochondrial DNA (mtDNA) content in large EVs preparations (*n* = 5 biologically independent experiments). **d** Small and large EVs isolated from conditional media were visualized by negative stain electron microscopy (scale bars = 500 nm) (*n* = 3 biologically independent samples). **e** Representative electron microscopy images of large EVs from *Rab7*^*−/−*^ MEFs containing mitochondria (scale bars = 500 nm) (*n* = 2 biologically independent samples). **f** Representative Western blots of proteins in large EV fraction from *Rab7*^*−/−*^ MEFs after Proteinase K digestion (*n* = 4). **g** Representative histograms of a Nanosight nanoparticle tracking analysis of large and small EVs secreted by wild type (WT) and *Rab7*^*−/−*^ MEFs. **h** Volcano plot of proteins identified in large EV fractions from WT and *Rab7*^*−/−*^ MEFs. Proteins are plotted according to their –log10P values and log2 fold enrichment (WT/*Rab7*^*−/−*^). Red dots represents proteins that are significantly enriched in *Rab7*^*−/−*^ EVs by two-sided Student’s *t*-test, while blue dots represents proteins that are decreased. **i** Gene ontology (GO) enrichment analysis of EV proteins isolated from *Rab7*^*−/−*^ MEFs relative to wild type MEFs with top terms for cellular component and biological process plotted according to—log10False Discovery Rate (FDR). Data are mean ± SEM. In (**b**), (**c**), and (**i**), statistical analysis and *P* values are by two-sided Student’s *t*-tests. Source data are provided as a Source Data file.

Next, we investigated additional conditions that directs MVBs to the plasma membrane for secretion of EVs rather than to lysosomes for degradation. Because the small GTPase Rab7 facilitates fusion of the MVB with the lysosome^25,26^, we investigated the effect of Rab7-deficiency on the release of large and small EVs into conditioned media. Western blot analysis of EV fractions showed that *Rab7*^−/−^ MEFs had increased release of large and small EVs into conditioned media at baseline compared to WT MEFs (Fig. 2a, b and Supplementary Fig. 3a). The large EVs were also positive for the mitochondrial proteins Tim23 and MnSOD, as well as the ER protein Calreticulin, which increased only in the large EV fractions from *Rab7*^*−/−*^ MEFs (Fig. 2a, b and Supplementary Fig. 3b). Restoring Rab7 levels in *Rab7*^−/−^ MEFs reduced baseline secretion of large EVs (Supplementary Fig. 3c), while overexpression of the Rab7 dominant negative, Rab7T22N, in WT MEFs trended to enhanced baseline secretion of large EVs (Supplementary Fig. 3d). Treatment of *Rab7*^*−/−*^ MEFs with GW4869, a known inhibitor of EV/exosome release^31^, diminished the release of mitochondria-containing large EVs from *Rab7*^*−/−*^ MEFs (Supplementary Fig. 3e). We further verified increased levels of entire mitochondria in the large EV fraction by quantifying mitochondrial DNA (mtDNA) inside the vesicles (Fig. 2c). Moreover, negative staining electron microscopy (EM) analysis confirmed size and spherical morphology of the isolated small and large EVs (Fig. 2d). Furthermore, transmission electron microscopy (TEM) showed the presence of mitochondria in large EVs isolated from conditioned media of *Rab7*^*−/−*^ MEFs (Fig. 2e). Proteinase K treatment of large EVs secreted by *Rab7*^*−/−*^ MEFs verified that Tsg101 and Tom20 were protected by a membrane while the exposed CD81 was digested (Fig. 2f and Supplementary Fig. 3f). Nanoparticle tracking analysis (NTA) showed the size distribution of large and small EVs released by WT and *Rab7*^*−/−*^ MEFs (Fig. 2g). The small EVs ranged from 50–560 nm with a large peak at 120–160 nM, whereas the large EV fraction spanned 115–550 nM with several peaks. Additionally, cargo proteins associated with large EVs secreted by WT and *Rab7*^*−/−*^ cells were identified by LC-MS/MS proteomics analysis. We identified 103 proteins that were significantly enriched in the large EV fraction from *Rab7*^*−/−*^ MEFs, including a variety of mitochondrial and ER proteins (Fig. 2h and Supplementary Data 1). Gene Ontology (GO) analyses also confirmed an enrichment of proteins associated with organelles and various metabolic processes in the cellular component and biological process categories, respectively, in large EVs isolated from *Rab7*^*−/−*^ cells (Fig. 2i). Taken together, these results suggest that Rab7-deficiency leads to increased release of large and small EVs from cells.

**Fig. 3:**
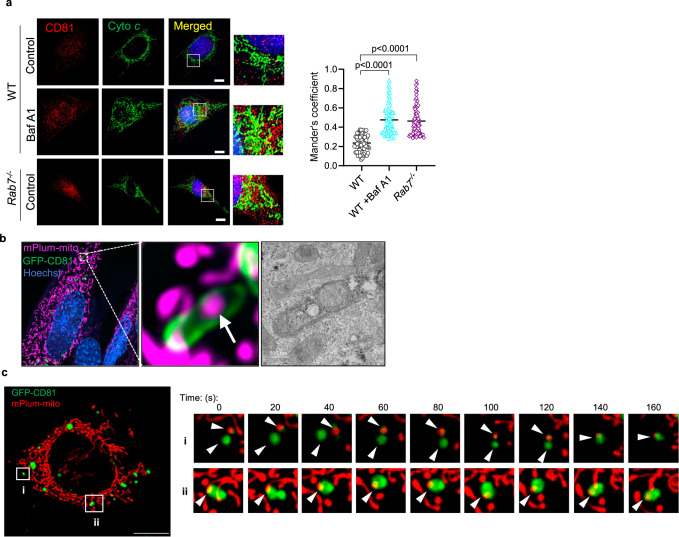
Sequestration of mitochondria in CD81-positive vesicles. **a** Co-localization between endogenous CD81 (red) and Cytochrome *c* (green) in WT +/− Baf A1 (50 nM for 24 h) and *Rab7*^*−/−*^ MEFs. Scale bar = 20 μm. Mander’s coefficient (*n* = 3 independent experiments with a total of 76 cells per condition). **b** Correlative light and electron microscopy of *Rab7*^*−/−*^ MEFs transfected with mPlum-mito3 and CD81-GFP (representative images are from *n* = 2). Fluorescent image and electron micrograph of a CD81-positive vesicle (green) containing a mitochondrion (magenta) are shown enlarged. **c** Time-lapse images from live cell imaging of *Rab7*^*−/−*^ MEFs overexpressing CD81-GFP (green) and mPlum-mito3 (red) (See Supplementary Movie 1). Arrowheads mark vesicles and mitochondria. Data are mean ± SEM. *P* values shown are by ANOVA with Tukey’s post-hoc testing. Source data are provided as a Source Data file.

Analysis of various proteins involved in regulating endosome function and trafficking showed that CD81 and Alix protein levels were significantly increased in *Rab7*^*−/−*^ MEFs (Supplementary Fig. 4a). *Rab7*^*−/−*^ cells also had modest but significantly increased levels of Rab5 (early endosomes), Rab4 (recycling endosomes), Rab11 (recycling endosomes), and Rab9 (Golgi-derived vesicles) (Supplementary Fig. 4b). Moreover, immunofluorescence analysis of CD81-positive vesicles in WT and *Rab7*^*−/−*^ MEFs confirmed increased co-localization between CD81-positive vesicles and mitochondrial Cytochrome *c* in *Rab7*^*−/−*^ MEFs at baseline and in WT cells after treatment with Baf A1 (Fig. 3a). Restoring Rab7 in *Rab7*^*−/−*^ MEFs reduced the co-localization between CD81-positive vesicles and Cytochrome *c* to levels observed in WT MEFs (Supplementary Fig. 4c). CD63 is another tetraspanin protein that is commonly associated with EV membranes, but CD63-positive vesicles rarely co-localized with mitochondria in WT and *Rab7*^*−/−*^ MEFs (Supplementary Fig. 4d). This suggest that distinct subpopulation of vesicles with cargo specificity might exist which is consistent with previous studies^32^. The presence of mitochondria in CD81-positive vesicles in *Rab7*^*−/−*^ MEFs was also confirmed by correlative light and electron microscopy (Fig. 3b). Live-cell imaging showed recruitment of GFP-CD81-positive vesicles to mitochondria (Fig. 3c and Supplementary Movie 1). Overall, these findings suggest that endosomal activity is enhanced in Rab7-deficient cells and that the Rab7-deficiency leads to sequestration of mitochondria into CD81-positive vesicles for secretion.

### Secretion of large EVs can occur independently of autophagy genes and PINK1

**Fig. 4:**
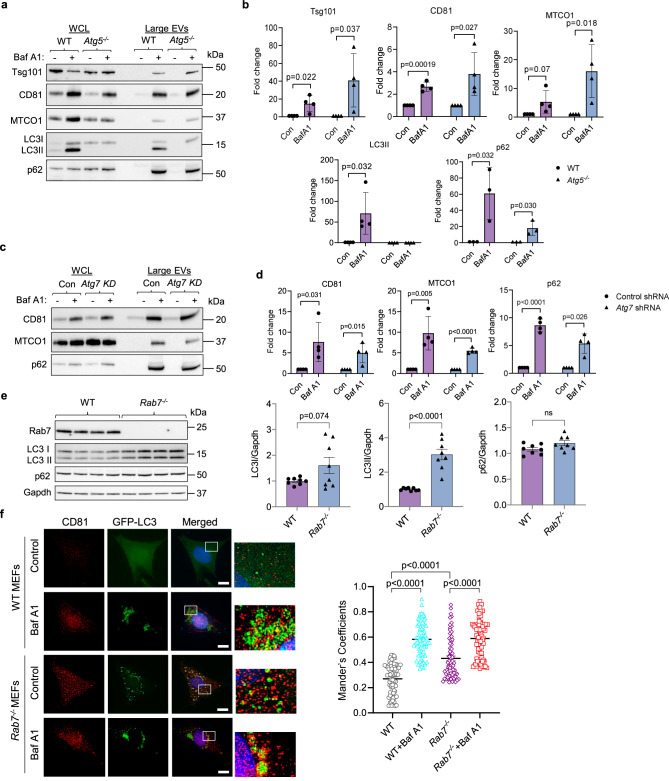
Secretion of large EVs is independent of the autophagy pathway. **a** Representative Western blots of Alix, Tsg101, CD81, MTCO1, LC3 and p62 protein levels in total cell lysates and EV preparations from WT and *Atg5*^*−/*−^ MEFs. The EVs were obtained by differential centrifugation of conditioned media collected over 48 h after treatment with vehicle or Bafilomycin A1 (Baf A1, 5 nM). EVs from 1 × 10^7^ cells were loaded. **b**. Quantification of proteins in large EVs (*n* = 4 biologically independent experiments). **c** Representative Western blot analysis of CD81 and MTCO1 protein levels in whole cell lysates (WCL) and EV fractions from MEFs (Control shRNA vs Atg7 shRNA) after treatment with vehicle or Bafilomycin A1 (Baf A1, 5 nM). **d** Quantification of proteins in large EV fractions (*n* = 4 biologically independent experiments). **e** Representative Western blots and quantification of LC3I, LC3II and p62 protein levels in WT and *Rab7*^*−/−*^ MEFs (*n* = 8 biologically independent experiments). **f** Representative images of co-localization between CD81 (red) and GFP-LC3 (green) in WT and *Rab7*^*−/*−^ MEFs +/− Baf A1 (50 nM for 24 h). Mander’s correlation coefficient (*n* = 3 independent experiments with a total of 71 WT and *Rab7*^*−/*−^ cells scored for controls and 74 cells scored for WT and *Rab7*^*−/−*^ treated with Baf A1). Scale bar = 20 μm. Data are mean ± SEM. ns = not significant. *P* values shown are by two-sided Student’s *t*-test (**b**, **d**, **e**) or ANOVA with Tukey’s post-hoc testing (**f**). Source data are provided as a Source Data file.

Some studies have reported that secretion of certain vesicles from cells is dependent on autophagosome formation^20,23^. Therefore, we examined whether secretion of mitochondria in large EVs required functional autophagy by evaluating EV release in WT and autophagy-deficient *Atg5*^*−/−*^ MEFs (Supplementary Fig. 5a). Despite impaired autophagosome formation, the presence of Baf A1 caused a significant increase in the release of large EVs containing mitochondria from *Atg5*^*−/−*^ MEFs (Fig. 4a, b). Interestingly, the large EV fraction from WT cells was also positive for LC3II, a protein presents on the autophagosome membrane^33^, and the autophagy adaptor protein p62/SQSTM1. Similar to Atg5-deficient cells, knockdown of *Atg7* in MEFs had no effect on EV secretion induced by Baf A1 treatment in MEFs (Fig. 4c, d and Supplementary Fig. 5b). To determine if mitochondrial secretion in EVs was dependent on functional mitophagy, we evaluated EV release in MEFs where PINK1 had been knocked down (KD) in MEFs (Supplementary Fig. 5c). However, cells with PINK1 knockdown had similar levels of CD81 and COX4s1 in the large EV fraction (Supplementary Fig. 5d, e), indicating that secretion of mitochondria can occur independently of Atg5/Atg7 and PINK1.

Rab7 facilitates fusion between autophagosomes and lysosomes, and Western blot analysis confirmed that *Rab7*^*−/−*^ MEFs have significantly elevated levels of LC3II compared to WT MEFs (Fig. 4e), an indication of autophagosome accumulation. Staining of cells with LysoTracker Red, which only accumulates in acidified lysosomes, suggested that lysosomes are still functional in *Rab7*^*−/−*^ MEFs (Supplementary Fig. 5f). Despite the accumulation of autophagosomes, levels of p62/SQSTM1 were unchanged in *Rab7*^*−/−*^ MEFs (Fig. 4e). Since p62 accumulates in cells when autophagy is impaired, unchanged p62 protein levels in *Rab7*^*−/−*^ MEFs suggests that an alternative mechanism of disposal might exist for p62-bound cargo. Autophagosomes have also been reported to fuse with endosomes to form hybrid structures known as amphisomes^34^. Therefore, we investigated whether disrupting lysosomal acidification or Rab7-deficiency induced fusion between autophagosomes and MVB. Immunofluorescence analysis showed increased co-localization between GFP-LC3 and CD81 in *Rab7*^*−/−*^ cells at baseline (Fig. 4f), indicative of amphisome formation^34^. Co-localization between GFP-LC3 and Cytochrome *c* was also increased in *Rab7*^*−/−*^ cells at baseline and was further enhanced after Baf A1 treatment (Supplementary Fig. 5g). The co-localization between GFP-LC3 and CD81 was also increased by Baf A1 treatment in WT and *Rab7*^*−/−*^ MEFs, suggesting a link between lysosomal dysfunction and amphisome formation. Taken together, these results demonstrate that secretion of large EVs is independent of autophagy. However, autophagosomes can fuse with MVBs to form amphisomes which might function to limit accumulation of autophagosomes containing cytotoxic cargo and allow for elimination of their cargo via secretion in EVs.

### Abrogation of EV secretion in *Rab7*^*−/−*^ MEFs enhances susceptibility to stress

**Fig. 5:**
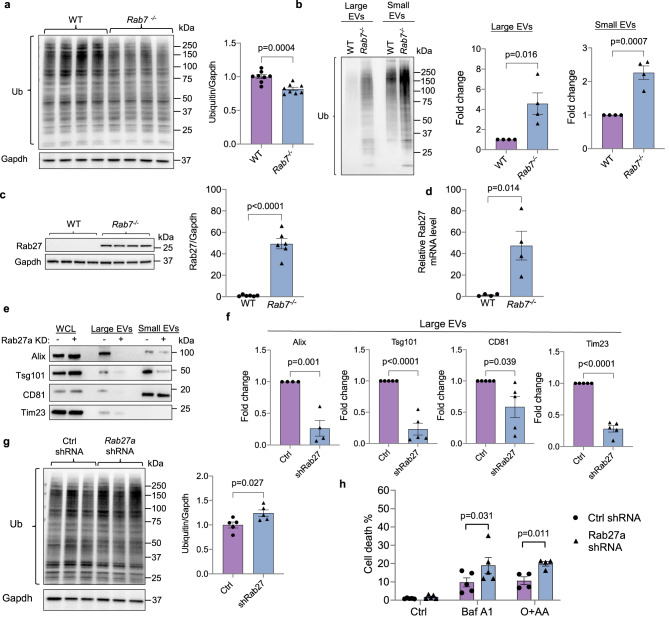
Rab27a knockdown in Rab7-deficient cells abrogates secretion of large EVs containing mitochondria and increases susceptibility to stress. **a** Representative Western blot and quantification of ubiquitinated proteins in WT and *Rab7*^*−/−*^ MEFs at baseline (*n* = 8 biologically independent experiments). **b** Representative Western blot and quantification of total ubiquitin levels in small and large EVs secreted by WT and *Rab7*^*−/−*^ MEFs at baseline (*n* = 4 biologically independent experiments). **c** Representative Western blot and quantification of Rab27a protein levels in WT and *Rab7*^*−/−*^ MEFs (*n* = 6 biologically independent experiments). **d** Rab27a transcript levels in WT and *Rab7*^*−/−*^ MEFs (*n* = 4 biologically independent experiments). **e** Western blot of Alix, Tsg101, CD81, and Tim23 protein levels in cell lysates and EV preparations from *Rab7*^*−/−*^ MEFs infected with control or Rab27a shRNA. **f** Quantification of proteins in large EVs (Alix *n* = 4; Tsg101, CD81, Tim23 *n* = 5 biologically independent experiments). **g** Western blot and quantification of ubiquitinated proteins in *Rab7*^*−/−*^ MEFs after Rab27a knockdown (*n* = 5 biologically independent experiments). **h** Analysis of cell death using Yo-Pro1 in *Rab7*^*−/−*^ MEFs infected with control or Rab27a shRNA. Cells were treated with Baf A1 (50 nM) for 24 h or O + AA (10 μM + 10 μM) for 24 h (Ctrl, Baf A1 *n* = 5, O + AA *n* = 4 biologically independent experiments). Data are mean ± SEM. n.s. not significant. *P* values shown are by two-sided Student’s *t*-test. Source data are provided as a Source Data file.

Autophagy is important in clearing ubiquitinated cargo that has been labeled for degradation^2^. Despite the impairments in autophagy, we observed a small decrease in ubiquitinated proteins in *Rab7*^*−/−*^ MEFs (Fig. 5a). It is currently unclear why disruption of internal degradation led to a decline in total ubiquitination when an increase would be expected with the impaired internal degradation. However, we detected increased levels of ubiquitinated proteins in both large and small EVs secreted by *Rab7*^*−/−*^ MEFs (Fig. 5b), suggesting that the alternative vesicular pathway functions to dispose ubiquitinated cargo that would otherwise be degraded inside the cell. This is also consistent with the lack of p62 accumulation in these cells as p62 binds to ubiquitinated cargo.

Rab27a is an important regulator of EV secretion and facilitates docking of MVBs to the plasma membrane^35^. Strikingly, we discovered that Rab27a protein and mRNA levels were significantly increased ~45-fold in *Rab7*^*−/−*^ MEFs compared to WT cells (Fig. 5c, d). Rab27a protein levels did not change in cells or hearts after pharmacological disruption of lysosomal function (Supplementary Fig. 6a, b). Knockdown of Rab27a in *Rab7*^*−/−*^ MEFs abrogated the release of both large and small EVs (Fig. 5e, f and Supplementary Fig. 6c, d), and was associated with a small but significant increase in levels of ubiquitinated proteins (Fig. 5g), supporting the notion that EVs release facilitates elimination of ubiquitinated cargo from cells. The disruption of EV release through knockdown of Rab27a did not affect mitochondrial content or mitochondrial biogenesis (Supplementary Fig. 6e, f). Finally, to examine if inhibiting EV release in *Rab7*^*−/−*^ MEFs alters susceptibility to stress, we treated cells with Baf A1 to disrupt lysosomal acidification or with Oligomycin+Antimycin A (O + AA) to inhibit mitochondrial respiration. Loss of Rab27a-mediated EV release exacerbated cell death in *Rab7*^*−/−*^ MEFs following either treatment (Fig. 5h), suggesting that this is an important mechanism in limiting accumulation of cytotoxic material in cells.

### Genetic ablation of *Rab7* in the adult mouse heart leads to increased secretion of EVs into cardiac tissue

**Fig. 6:**
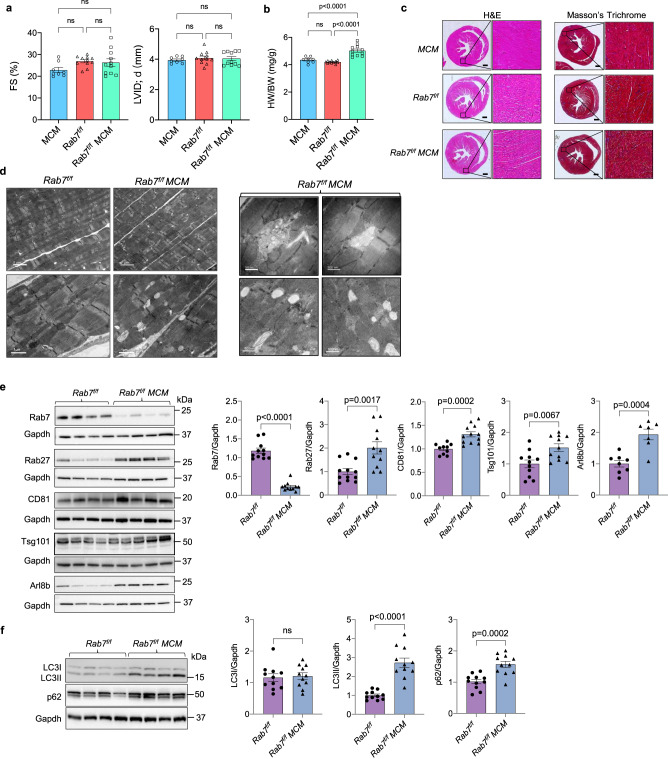
Characterization of mice with cardiac specific deletion of *Rab7*. **a** Echocardiographic analysis of ventricular function and structure at 28 days post-tamoxifen treatment. Percent (%) fractional shortening (FS) and left ventricular internal dimension in end diastole (LVID;d). *MCM* (*n* = 8 biologically independent animals), *Rab7*^*f/f*^ (*n* = 11 biologically independent animals), *Rab7*^*f/f*^ *MCM* (*n* = 11 biologically independent animals). **b** Heart weight to body weight ratio (HW/BW). *MCM* (*n* = 8), *Rab7*^*f/f*^ (*n* = 11 biologically independent animals), *Rab7*^*f/f*^ *MCM* (*n* = 11 biologically independent animals). **c** Hematoxylin and eosin (H&E) and Masson’s Trichrome staining of heart sections. Scale bar = 0.5 mm. **d** Ultrastructural analysis by transmission electron microscopy at D28 (representative of *n* = 2 biologically independent samples). **e** Representative Western blots and quantification of endosomal proteins (Rab7/Gapdh: *Rab7*^*f/f*^ (*n* = 11 biologically independent animals) *Rab7*^*f/f*^ *MCM* (*n* = 11 biologically independent animals); Rab27/Gapdh: *Rab7*^*f/f*^ (*n* = 12 biologically independent animals), *Rab7*^*f/f*^ *MCM* (*n* = 11 biologically independent animals); CD81/Gapdh: *Rab7*^*f/f*^ (*n* = 10 biologically independent animals), *Rab7*^*f/f*^ *MCM* (*n* = 12); Tsg101/Gapdh: *Rab7*^*f/f*^ (*n* = 11 biologically independent animals), *Rab7*^*f/f*^ *MCM* (*n* = 11 biologically independent animals); Arl8b/Gapdh: *Rab7*^*f/f*^ (*n* = 8 biologically independent animals), *Rab7*^*f/f*^ *MCM* (*n* = 7 biologically independent animals)). **f** Representative Western blots and quantification of autophagy proteins (*n* = 11 biologically independent animals). Data are mean ± SEM. ns = not significant. *P* values shown are by ANOVA with Tukey’s post-hoc testing (**a**) or two-sided Student’s *t*-test (**e**, **f**). Source data are provided as a Source Data file.

To investigate the role of Rab7 in regulating EV release in vivo, we crossed *Rab7*^*f/f*^ mice with the tamoxifen-inducible Myh6-MerCreMer (*MCM*) transgenic mice to allow for myocyte-specific deletion of *Rab7* in the adult heart^36^. *MCM, Rab7*^*f/f*^ and *Rab7*^*f/f*^ *MCM* mice were injected with 5 doses of tamoxifen and cardiac function was evaluated 28 days later (Supplementary Fig. 7a). Echocardiographic assessment revealed no significant changes in left ventricular contractile function or structure (Fig. 6a and Supplementary Fig. 7b). Gross examination of hearts revealed a small but significant increase in heart weight to body weight (HW/BW) ratio in *Rab7*^*f/f*^ *MCM* mice compared to *MCM and Rab7*^*f/f*^ mice (Fig. 6b), suggesting development of mild cardiac hypertrophy. However, histological analysis of hearts demonstrated normal cardiac structure with no fibrosis in *Rab7*^*f/f*^ MCM mice (Fig. 6c). Mitochondrial respiration was also normal in *Rab7*^*f/f*^ *MCM* hearts (Supplementary Fig. 7c). Evaluation of changes at the ultrastructural level by TEM confirmed normal myofibrillar structure and mitochondria but revealed the presence of many small vacuoles in Rab7-deficient myocytes (Fig. 6d).

Next, we examined whether loss of Rab7 in the heart was associated with changes in proteins involved in regulating endosomal trafficking and EV secretion. We found that Rab27, CD81, Tsg101 and Arl8b were significantly increased in Rab7-deficient hearts (Fig. 6e). Because Rab7 functions in the autophagy pathway, we also examined the effect of *Rab7* deletion on autophagic activity in hearts. Consistent with our in vitro findings, autophagic flux was impaired in Rab7-deficient hearts as assessed by significant accumulation of LC3II and the autophagy adaptor protein p62 (Fig. 6f).

**Fig. 7:**
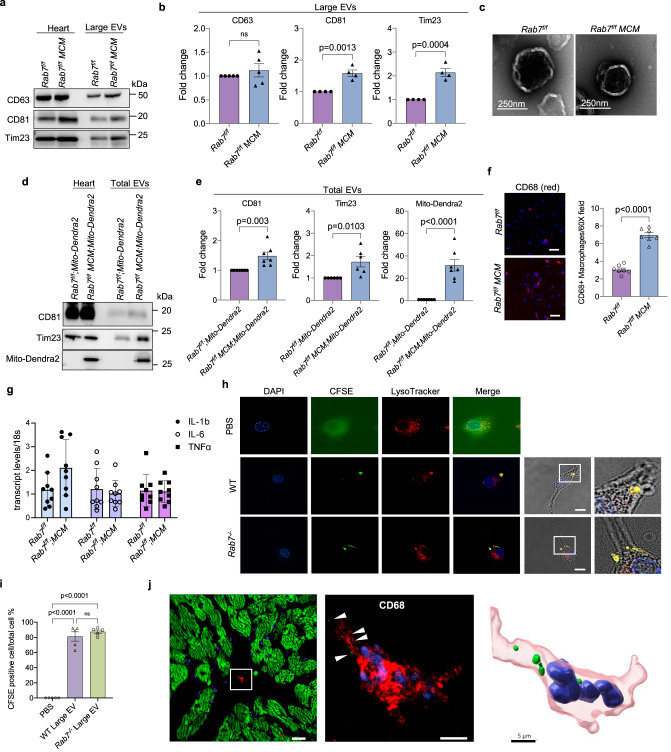
Enhanced secretion of large EVs containing mitochondria in Rab7-deficient hearts. **a** Representative Western blots of CD63, CD81 and Tim23 in whole heart lysates and large EV fractions from *Rab7*^*f/f*^ and *Rab7*^*f/f*^ *MCM* heart tissue. **b** Quantification of protein levels in large EV fractions (CD63: *Rab7*^*f/f*^ (*n* = 5 biologically independent animals) *Rab7*^*f/f*^ *MCM* (*n* = 5 biologically independent animals); CD81: *Rab7*^*f/f*^ (*n* = 4 biologically independent animals), *Rab7*^*f/f*^ *MCM* (*n* = 4 biologically independent animals); Tim23: *Rab7*^*f/f*^ (*n* = 4 biologically independent animals), *Rab7*^*f/f*^ *MCM* (*n* = 4 biologically independent animals)). **c** Negative stain electron microscopy of large EVs isolated from *Rab7*^*f/f*^ and *Rab7*^*f/f*^ MCM heart tissue (representative of *n* = 3 biologically independent animals). **d** Representative Western blots of CD81, Tim23, and Mito-Dendra2 protein levels in whole heart lysates and extracellular vesicle fractions from *Rab7*^*f/f*^;*Mito-Dendra2* and *Rab7*^*f/f*^*MCM;Mito-Dendra2* heart tissue. Large and small (total) EVs were isolated from hearts using immunoaffinity capture method. **e** Quantification of protein levels in the EV fractions (CD81, MitoDendra2: *Rab7*^*f/f*^;*Mito-Dendra2* (*n* = 7 biologically independent animals), *Rab7*^*f/f*^*MCM;Mito-Dendra2* (*n* = 7 biologically independent animals); Tim23: *Rab7*^*f/f*^;*Mito-Dendra2* (*n* = 6 biologically independent animals), *Rab7*^*f/f*^*MCM;Mito-Dendra2* (*n* = 6 biologically independent animals)). **f** Immunostaining and quantification of CD68-positive (red) cells in *Rab7*^*f/f*^ and *Rab7*^*f/f*^ *MCM* hearts (*n* = 7 biologically independent animals), Scale bar = 20 μm. **g** qPCR analysis for inflammatory markers in *Rab7*^*f/f*^ and *Rab7*^*f/f*^ *MCM* hearts (*n* = 9 biologically independent animals). **h** Assessment of large EV uptake by RAW 264.7 macrophages. Representative images of cells after incubation (24 h) with CFSE-labelled EVs (green). Blue (nuclei) and red (Lysotracker Red). Scale bar = 10 μm. **i** Quantification of CFSE-labeled EV uptake by cells (*n* = 5, a minimum of 100 cells were scored in each independent experiment). **j** Frozen heart section from *Rab7*^*f/f*^ *MCM*; *Mito-Dendra2* mice shows co-localization between Mito-Dendra2 (green) and a CD68-positive macrophage (red) (white arrow heads) (representative of *n* = 6 biologically independent samples). 3D reconstruction from confocal image illustrates the presence of Mito-Dendra2-positive mitochondria (green) from myocytes inside the macrophage (red). Scale bars = 10 and 5 μm. Data are mean ± SEM. ns = not significant. *P* values shown are by two-sided Student’s *t*-test (**b**, **e**, **f**, **g**) or ANOVA with Tukey’s post-hoc testing (**i**). Source data are provided as a Source Data file.

Because mitochondrial respiration and cardiac function were normal in Rab7-deficient hearts despite impaired autophagy, we investigated whether loss of Rab7 in the heart led to enhanced secretion of EVs. Indeed, Rab7-deficient hearts had increased levels of large EVs in the cardiac tissue (Fig. 7a, b and Supplementary Fig. 8a), whereas *MCM* mice had similar EV release as WT (Supplementary Fig. 8b, c). Negative staining EM confirmed size and spherical morphology of large EVs isolated from the *Rab7*^*f/f*^ and *Rab7*^*f/f*^ *MCM* heart tissues (Fig. 7c) while NTA showed that vesicle sizes ranged from ~60 nm to ~530 nm (Supplementary Fig. 8d). We also found increased levels of the mitochondrial protein Tim23 as well as elevated mtDNA content in large EVs isolated from *Rab7*^*f/f*^ *MCM* hearts (Fig. 7a, b and Supplementary Fig. 8e). To further confirm that the mitochondria detected in EVs originated from cardiac myocytes, we isolated total EVs (large and small) from cardiac tissue of *Rab7*^*f/f*^; *Mito-Dendra2* and *Rab7*^*f/f*^ *MCM*; *Mito-Dendra2* mice using immunoaffinity purification. Western blot analysis of EVs isolated from hearts of *Rab7*^*f/f*^ *MCM*; *Mito-Dendra2* mice showed that they were positive for both Tim23 and Mito-Dendra2 (Fig. 7d, e), confirming that the mitochondria originated from cardiac myocytes. Altogether, these results demonstrate that cardiac specific Rab7-deficiency leads to impaired autophagic degradation concurrent with increased release of large EVs containing mitochondria in the heart.

Similar to CQ treatment of WT mice, the levels of CD81-positive EVs circulating in plasma did not increase after deletion of *Rab7* in the adult heart (Supplementary Fig. 8f), suggesting that the secreted EVs remain in the cardiac tissue. Therefore, we investigated the fate of released EVs in *Rab7*^*f/f*^ *MCM* mice. The primary function of macrophages is to dispose unwanted material through phagocytosis^37^. Immunofluorescent staining of heart sections and flow cytometry showed that Rab7-deficient hearts had a significant increase in cardiac macrophages (Fig. 7f and Supplementary Fig. 8g), however, no transcriptional changes were observed in inflammatory markers (Fig. 7g). To assess if macrophages take up large EVs, carboxyfluorescein succinimidyl ester (CFSE)-labelled EVs isolated from conditioned media of WT or *Rab7*^*−/−*^ MEFs were added to cultured RAW 264.7 macrophages. After 24 h of incubation, a majority of cells were positive for CFSE (Fig. 7h, i), and the CFSE fluorophore co-localized with LysoTracker Red, indicating delivery of EVs to lysosomes after uptake. Finally, 3D imaging analysis of heart sections prepared from *Rab7*^*f/f*^ *MCM*; *Mito-Dendra2* mice confirmed the presence of Mito-Dendra2 (green)-positive mitochondria inside a CD68-positive macrophage (red) (Fig. 7j and Supplementary Movie 2). Thus, loss of Rab7 in adult myocytes amplifies EV release during autophagy dysfunction and this response is sufficient to maintain cardiac homeostasis in vivo as cardiac macrophages ensure phagocytosis of released cargo.

### Increased secretion of EVs in hearts with age and in Danon disease

**Fig. 8:**
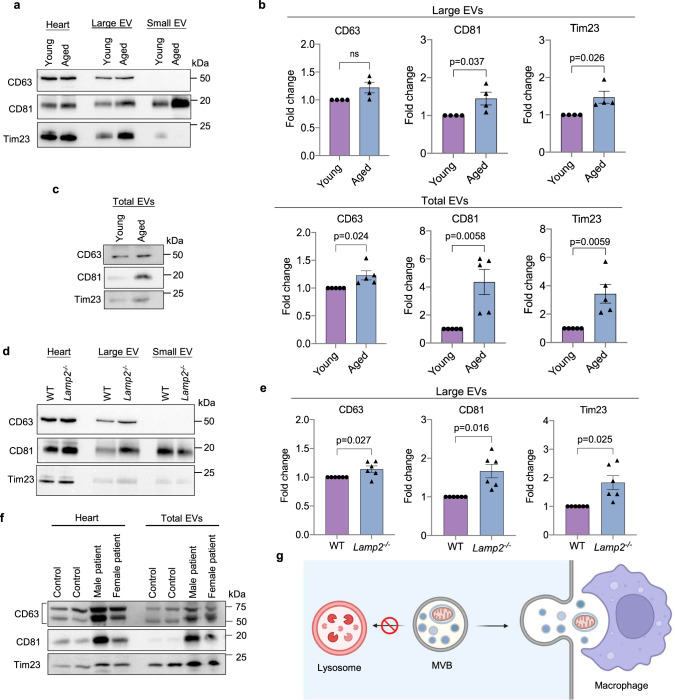
Enhanced secretion of large EVs containing mitochondria in aged and *Lamp2*-deficient hearts. **a** Representative Western blots of CD63, CD81, and Tim23 in whole heart lysates and cardiac tissue EVs prepared from young (4 month) and aged (24 month) mice using differential centrifugation. **b** Quantification of proteins in large EV fractions (*n* = 4 biologically independent animals). **c** Representative Western blots and protein quantification of total (large and small) EVs in hearts using immunoaffinity capture (*n* = 5 biologically independent animals). **d** Representative Western blots of CD63, CD81 and Tim23 in whole heart lysates and cardiac tissue EVs prepared from WT and *Lamp2*^*−/−*^ mice using differential centrifugation. **e** Quantification of proteins in large EV fractions (*n* = 6 biologically independent animals). **f** Western blot of heart lysates and cardiac tissue EVs prepared from control and Danon Disease patients (male and female). Data obtained from four independent donors. **g** Overview of EV secretion in cells (Created with BioRender.com). Data are mean ± SEM. ns = not significant. *P* values shown are by two-sided Student’s *t*-test. Source data are provided as a Source Data file.

The burden on lysosomes to eliminate damaged proteins and organelles increases with age in long-lived cells such as cardiac myocytes. Studies have reported various age-related changes in lysosomes ranging from alterations in size and number to impaired lysosomal hydrolase activity^38^. To investigate if aging affects the release of EVs in vivo, we evaluated the level of EV secretion in hearts of young (4-month-old) and aged (24-month-old) WT mice. We observed a significant increase in levels of large EVs containing mitochondria in cardiac tissue from aged mice using differential centrifugation (Fig. 8a, b) or immunoaffinity purification (Fig. 8c). A loss-of-function mutation in *LAMP2* is known to underlie Danon disease which is associated with a defect in autophagic-lysosomal-mediated degradation. To investigate if Lamp2-deficiency in mice correlated with increased EV secretion, we isolated large EVs from hearts of WT and *Lamp2*^*−/−*^ mice at 4 months of age, prior to any cardiac dysfunction^27,39^. Western blot analysis showed increased levels of large EVs containing mitochondria in *Lamp2*^*−/−*^ hearts (Fig. 8d, e). The levels of small EVs in cardiac tissue did not increase in aged hearts or in *Lamp2*-deficiency (Supplementary Fig. 9a, b). Finally, we analyzed EV content in cardiac biopsies obtained at the time of explant from two Danon Disease patients, a young male (17 years) who underwent total artificial heart implantation and a female (39 years) who underwent heart transplantation. Immunoaffinity capture of EVs (large and small) using bead conjugated antibodies against EV cell surface markers showed that the Danon Disease patients had higher levels of EVs positive for mitochondria in cardiac tissue compared to normal control hearts (Fig. 8f). Collectively, these results confirm that a compromise in internal degradation pathways due to age or genetic mutations leads to increased secretion of mitochondria-containing EVs from cells.

## Discussion

Mitochondria play a central role in cardiac energy metabolism and are responsible for generating most of the energy needed to sustain contraction. Because cardiac myocytes are post-mitotic, maintaining a healthy population of mitochondria is essential for their long-term survival. Intracellular degradation pathways that are responsible for eliminating defective and potentially harmful mitochondria rely on lysosomes for the breakdown of cargo. In this study, we identified an alternative pathway for elimination of mitochondria and ubiquitinated proteins from cells when lysosomal function is compromised that involves their secretion in large EVs (Fig. 8g). These EVs are positive for markers found on traditional exosomes/small EVs suggesting that they originate in late endosomal compartments that fuse with the plasma membrane to release the intralumenal vesicles into the extracellular environment. Although this pathway is independent of autophagy, autophagosomes can deliver cargo to CD81-positive vesicles through fusion to form an amphisome. Our findings also demonstrate that loss of the small GTPase Rab7 leads to increased secretion of large EVs. The secreted large EVs do not enter the circulation to a significant extent but undergo phagocytosis by adjacent macrophages for delivery to lysosomes. Overall, these findings demonstrate that secretion of mitochondria in EVs can serve as an alternative pathway of elimination when internal degradation pathways are compromised.

It is well established that the internal pathways responsible for eliminating plasma membrane receptors, protein aggregates or organelles rely on lysosomes for degradation and recycling of cargo^9^. However, lysosomal function declines with age^38^ and acidification can be impaired by the chemotherapeutic drug doxorubicin^40^. Also, loss-of-function mutations in *LAMP2* leads to profound cardiac hypertrophy and heart failure in patients^28^. Although the penetrance of disease is nearly 100% in males, affected individuals are often asymptomatic in early childhood. Similarly, *Lamp2*^*−/−*^ mice accumulate autophagosomes due to impaired flux, but do not display left ventricular dysfunction until a later age^39^. This suggests that alternative mechanisms exist in the heart that may temporarily compensate for the defect in lysosomal function. Accordingly, our findings demonstrate that disrupting lysosomal function pharmacologically or genetically in cells and mice led to increased secretion of both small and large EVs from cells.

Specific studies on Rab7 in hearts are still limited but it has been reported that downregulation of Rab7 is associated with a defect in autophagy and activation of necrosis in cardiac-specific Csn8 knockout mice^41^. Rab7 has also been reported to provide protection against myocardial I/R by facilitating mitochondrial fission and mitophagy^42,43^. Considering these previous findings and Rab7’s key role in autophagy and vesicle trafficking, it was unexpected that conditional deletion of *Rab7* in the heart was well tolerated. Loss of Rab7 had little effect on mitochondrial health and cardiac function at baseline despite compromised autophagy and accumulation of vesicular structures. Instead, there was an increase in the release of EVs into the extracellular space of these hearts, suggesting that this might function as an alternative cellular quality control pathway to eliminate cytotoxic materials or to limit accumulation of autophagosomes. However, Rab7-deficiency led to an increase in cardiac mass at D28 which suggests that loss of *Rab7* will likely be detrimental to myocytes over time as well as increase the heart’s susceptibility to stress.

Cells can secrete many types of vesicles of various sizes and origins. For instance, adult neurons and cardiac myoyctes can extrude protein aggregates and mitochondria in large (3.5–4 μm) vesicular structures called exophers^19,20^. Similarly, mesenchymal stem cells can transfer mitochondria to macrophages by secretion in microvesicles^23^. This study observed that mitochondria sequested in autophagosomes are transported to the plasma membrane, where they are released by outward budding of the plasma membrane. Recent studies have reported that lysosomal inhibition can activate secretory autophagy to clear autophagic cargo and mitochondria from cells^44–46^. Tan et al. reported that clearance of mitochondria via the secretory autophagy pathway is independent of Atg5/7-mediated autophagosome formation^45^, while Solvik and colleagues showed that secretion of autophagic cargo adaptors, such as p62 and Optineurin, via secretory autophagy requires Atg proteins^44^. Here, we found that mitochondria and p62 can be secreted independently of Atg5 and Atg7 in vesicles that are similar to small EVs (i.e. exosomes), an indication that these vesicles originate from the same endosomal pathway. Our finding that disrupting autophagosome formation alone failed to increase EV secretion at baseline also suggests that alternative internal clearance pathways are compensating and delivering cargo to lysosomes for degradation. It also suggests that the EV secretion pathway is linked to lysosomal function and is primarily utilized when lysosomal function is compromised or overwhelmed.

Although the molecular mechanisms involved in regulating secretion of various vesicles are still uncertain, it is clear that cells have multiple multiple pathways in place for excretion of cargo. Also, extensive cross-talk exists and autophagosomes can utilize the EV secretion pathway to eliminate cargo from cells by fusing with endosomes to form hybrid organelle structures known as amphisomes^34^. Although the amphisome is thought to represent a transitional state prior to fusion with a lysosome, there is also evidence that this hybrid vesicle can fuse with the plasma membrane to release cargo^34^. Thus, it is possible that the fusion with MVB leads to gain of proteins involved in facilitating transport of the hybrid vesicle to the plasma membrane for fusion. Excessive accumulation of autophagosomes due to compromised fusion between autophagosomes and lysosomes can be cytotoxic and lead to cardiomyopathy^47^. Formation of the amphisome could represent a mechanism to facilitate secretion of cargo to limit autophagosome accumulation and reduce cardiotoxicity.

It is well established that EVs can function as signaling vesicles by delivering nucleotides and proteins to other cells^10^. Uptake of encapsulated mitochondria has also been reported to influence cellular function after uptake. For instance, microvesicles enriched in mitochondria released by monocytic cells are pro-inflammatory and induce type I Interferons (IFN) and Tumor necrosis factor (TNF) signaling in endothelial cells after uptake^22^, whereas mitochondria phagocytosed by macrophages undergo fusion with the existing mitochondrial network and enhance respiration^23^. Moreover, adipocytes release damaged mitochondrial fragments into circulation in small EVs, which are then taken up by tissues including the heart and liver. A burst of ROS is generated in myocytes after uptake, which seems to precondition the heart against future stress^21^. Although some secreted EVs can function in cell-to-cell communication, our data suggest that secretion of EVs in response to lysosomal impairement can function as an alternative quality control mechanism to dispose of cargo. Ubiquitination is an important post-translational modification involved in targeting proteins and organelles for degradation^48^. In our study, both large and small EVs contained p62 and ubiquitinated cargo, depolarization of mitochondria enhanced secretion of mitochondria in large EVs, and inhibiting EV release increased suceptibility to stress when internal degradation was compromised. Additionally, our observation that the phagocytosed EVs were delivered to lysosomes in macrophages is further evidence that this might function as an alternative disposal pathway. Interestingly, we did not observe a significant increase in circulating EVs in plasma of mice after 24 h of CQ treatment, suggesting that EVs were retained in tissues. It is possible that these EVs are not involved in cell-cell communication but contain cargo that is destined for disposal in lysosomes. Thus, degradation by resident tissue macrophages represents the most efficient method of disposal. Future studies should focus on investigating whether EVs involved in signaling versus cargo elimination contain specific cell surface markers that dictate their fate after secretion. In contrast to our finding, Solvik et al. found that levels of LC3 and p62 in plasma increase in mice after 3 days of CQ treatment^44^, suggesting that tissue vesicles might enter circulation with extended inhibition of lysosomal function.

Rab7 is known to be involved in regulating many different processes in cells, including trafficking of vesicles and organelles on microtubules both towards plus and minus ends^49^. However, how Rab7 regulates trafficking appears to be complex and depends on the type of vesicle. For example, our findings suggest that Rab7-deficiency diverts trafficking of LE/MVB to the plasma membrane for secretion of mitochondria-containing EVs. Rab7 is important for fusion between MVB and lysosomes and it is likely that a defect in this process due to lack of Rab7 diverts the MVBs to the plasma membrane. This also functions to prevent accumulation of MVB in the cell. A similar role for Rab7 has been reported in trafficking of the Hepatitis B virus that resides in MVB after infection. This study found that activation of Rab7 increases fusion between the MVB and lysosomes resulting in degradation of the virus, whereas depletion of Rab7 or disruption of lysosomal acidification enhances virus secretion from the cells^50^. Our findings that restoring Rab7 protein in *Rab7*^*−/−*^ MEFs decreased EV secretion whereas blocking Rab7 activity with a dominant negative protein increased EV secretion in WT cells also suggest that Rab7 activity might regulate trafficking of MVBs. However, it is also possible that some of the increase in EV secretion in *Rab7*^*−/−*^ MEFs is due to general trafficking defects rather than a direct regulatory function of Rab7. Other studies have reported that Rab7 is a positive regulator of EV secretion where single cell imaging experiments demonstrated that overexpression of Rab7 in HeLa cells leads to increased secretion of small CD63-positive EVs at baseline^51^. Another study reported that Rab7 regulates trafficking of vesicles containing newly synthesized transferrin receptor from the *trans* Golgi network to the plasma membrane^52^. Clearly, Rab7’s trafficking functions are complex and further studies are necessary to delineate whether its pleiotropic functions in vesicle trafficking rely on factors such as the type of vesicle and its cargo.

In sum, our study delineates a distinct process of mitochondrial quality control in cells and heart when lysosomal-mediated degradation is compromised. As mitochondria are of bacterial origin, secretion of encapsulated mitochondria avoids activation of a potentially harmful inflammatory response. There is currently a strong interest in developing therapeutics that activates autophagy in the heart. However, increasing autophagosome formation to levels that exceeds the capacity of lysosomal degradation could lead to increased secretion of cargo by the cells. Although the impact of EV secretion on surrounding cells appears to be minimal, future studies need to elucidate whether excessive secretion affects the function of other cell types in the heart.

## Methods

### Animals

All animal experiments were performed following the Guidelines of National Institutes of Health on the Use of Laboratory Animals and approved by the Institutional Animal Care and Use Committee at the University of California, San Diego.

*Rab7*^*f/f*^ mice^25^ were crossed with cardiac specific *Myh6-MerCreMer* (MCM) (Jackson Laboratory Stock #005650) to generate cardiomyocyte-specific *Rab7* knockout mice. Cre negative littermates were used as controls. To selectively delete *Rab7* in myocytes, male and female mice 8–10 weeks of age were injected (i.p.) with 40 mg/kg tamoxifen (Sigma-Aldrich, T5648) for five consecutive days. Control mice were also injected with tamoxifen.

Mice with cardiac specific expression of mito-Dendra2 were generated by breeding female *PhAM-floxed*^53^ (Jackson Laboratory, Stock #018385) and male *Myh6-Cre* or *Myh6-MerCreMer* mice. *Lamp2* knockout mice have previously been described^54^. Young (4-month-old) and Aged (24-month-old) male C57BL/6 mice were obtained from the National Institute of Health Institute of Aging colony (Charles River). For the in vivo chloroquine injection, saline or 80 mg/kg chloroquine (Sigma-Aldrich, C6628) was administered to mice by intraperitoneal injection, and the heart tissue were harvested 24 h later.

### Echocardiography

Transaortic echocardiography was performed using a Vevo 3100LT equipped with MX 400 transducer (30 MHz) for small animals as previously reported^55^. Mice were kept on a circulating water pad (37 °C) and maintained under light anesthesia (0.5%–1% isoflurane, 98%–99.5% O2) during the procedure. M- and B-mode views were acquired, and cardiac function was analyzed using the VisualSonics software.

### Histological Analysis

To prepare paraffin sections, hearts were rinsed in PBS, fixed in 10% neutral buffered formalin for 48–72 h and then transferred to 70% dehydrant alcohol for 48 h. Hearts were embedded in paraffin, cut into 5-μm sections using a microtome (Leica Biosystems) and stained with hematoxylin and eosin (H&E) or Masson’s trichrome (Sigma-Aldrich). To prepare frozen sections, hearts were rinsed in PBS and then fixed in 10% neutral buffered formalin (Sigma) for 48–72 h at 4 °C followed by incubation in 15% sucrose (6 h) and 30% sucrose (12 h) solutions. Hearts were frozen in Tissue-Tek® O.C.T. Compound (SAKURA) for sectioning by a cryostat.

For fluorescence staining, frozen heart sections prepared from *Rab7*^*f/f*^*, Rab7*^*f/f*^ *MCM, Rab7*^*f/f*^ *MCM; Mito-Dendra2* mice were incubated with anti-CD68 (Invitrogen 14-0681-82, 1:100 dilution) overnight and then with goat anti-rat secondary antibody (Thermo Fisher Scientific, A-11007, 1:200 dilution) for a duration of 90 min at room temperature. Stained sections were mounted using VECTASHIELD HardSet Mounting Media with DAPI (Vector Laboratories) and images were captured using Nikon Eclipse microscope. ImageJ was used to quantify the number of CD68-positive cells in *Rab7*^*f/f*^ *and Rab7*^*f/f*^ *MCM* heart sections.

### Mitochondrial respiration measurements

Mitochondria were isolated from *Rab7*^*f/f*^ and *Rab7*^*f/f*^ *MCM* mouse hearts by investigators blinded to the genotype, and mitochondrial respiration was measured using the Seahorse XP Flux Analyzer (Seahorse Bioscience – Agilent Technologies) following a modified protocol described previously^56^. Data were analyzed using Wave for Desktop (Seahorse Bioscience – Agilent Technologies).

### Cell culture, adenoviral infection, and lentiviral transduction

WT and *Atg5*^*−/−*^ mouse embryonic fibroblast (MEF) were obtained from Dr. Noboru Mizushima (The University of Tokyo, Japan). WT and *Rab7*^*−/−*^ MEFs were generously provided by Dr. Aimee Edinger (UC Irvine). Cells were cultured in Dulbecco’s Modified Eagle Medium (DMEM) containing 10% Fetal Bovine Serum (FBS), 1% Antibiotic-Antimycotic supplement at 37 °C in 5% CO2. Raw 264.7 macrophages were cultured in RPMI 1640 containing 10% FBS, 1% antibiotic-antimycotic supplement at 37 °C in 5% CO2. Cell lines were routinely tested for mycoplasma. MEFs were infected with adenoviruses encoding GFP-Rab7 or GFP-Rab7T22N in DMEM + 2% heat inactivated serum for 3 h and then rescued with growth media. Experiments to collect EVs were initiated 24–36 h post-infection. Rab27, Atg7 and PINK1 knockdowns were achieved using the MISSION® shRNA system. The shRNA lentiviral transduction particles were purchased from Millipore-Sigma to deliver and stably express shRNAs in MEFs. MISSION® pLKO.1-puro Non-Mammalian shRNA Control Transduction Particles (#SHC002V), MISSION shRNA Lentiviral Transduction Particles (mouse) for RAB27A (#HCLNVNM-TRCN0000100577), RAB7 (#SHCLNV-TRCN0000100883), ATG7 (#SHCLNV-TRCN0000375444), and PINK1 (#SHCLNV-TRCN0000026750). The cells were transduced with lentiviral particles (2 MOI) for 48 h at 37 °C with polybrene (8 µg/ml) followed by puromycin (3 μg/ml) selection for 48–72 h.

### Isolation of EVs

Large and small EVs were isolated using a modified differential centrifugation protocol as previously described^29^. Total EVs (small and large) were isolated for experiments shown in Figs. 7d, e, 8c, f, and Suppl. Figs. 2b, 2b, and 8f. Briefly, MEFs were seeded at density of 3 × 10^5^ cells/ml in a T182 flask and cultured in DMEM containing 10% EV-depleted FBS (Gibco) for 24–48 h prior to collection of conditioned media. In some experiments, dimethyl sulfoxide (DMSO) or Bafilomycin A1 (5 nM) were added to the cells after plating. The conditioned media was subjected to centrifugation at 300 × *g* and 2000 × *g* (10 min each at 4 °C) to remove unattached cells and large debris. The resulting supernatant was centrifuged at 10,000 × *g*, 4 °C for 50 min to pellet large EVs and the final supernatant was centrifuged in at 100,000 × *g* for 70 min to pellet small EVs. Large and small EV pellets were resuspended in Triton lysis buffer for Western Blot analysis or in PBS for nanoparticle tracking analysis (NTA) using NanoSight NS300.

To isolate EVs from mouse and human tissue, we utilized a previously published protocol^57^ with minor modifications. Briefly, heart tissues were cut into small pieces (2 × 2 × 2 mm) and then incubated for 45 min at 37 °C either in Hanks′ Balanced Salt solution (HBSS) or plain DMEM media supplemented with Liberase HD (200 μg/ml, Roche) and DNase I (20 U/ml, Roche) with mild agitation (50 rpm) to separate the EV from the cardiac tissue. After enzymatic digestion, dead cells and tissue debris were removed by filtration (70 µm filter) followed by successive centrifugation at 300 × *g* for 10 min and 2000 × *g* for 20 min. The resulting supernatant was filtered using 0.8 µm filter unit (GE Healthcare) to eliminate apoptotic bodies and larger vesicles such as exophers^20^. The filtered supernatant was centrifuged at 16,500 × *g* for 25 min and 118,000 × *g* for 2.5 h to collect large and small vesicles, respectively. Large and small EV pellets were resuspended in Triton lysis buffer for Western blot analysis or in PBS for NTA using NanoSight NS300.

Alternatively, tissue and plasma EVs (large or total EVs) were isolated using an immunoaffinity purification method where the EVs are purified based on their surface markers (i.e. CD9, CD63, CD81) using the Miltenyi Biotec Pan Kit (human and mouse). Briefly, large EV- and small EV-enriched pellets obtained from differential centrifugation were resuspended in 500 μl HBSS plus 50 μL of Exosome Isolation MicroBeads (Miltenyi Biotec) recognizing EV surface markers CD9, CD63 and CD81, and incubated overnight at 4 °C. Magnetic separation was performed and the EVs were eluted in Triton-X 100 lysis buffer for WB analysis.

Mouse plasma samples were collected in K3 EDTA tube (Sarstedt Inc) and plasma were separated by centrifugation at 2000 × *g* for 15 min, followed by diluting with equal volume of PBS and centrifuged at 2000 × *g* for 30 min to remove cell debris and larger vesicles. The supernatant was incubated with MicroBeads (Miltenyi Biotec Pan Kit, mouse), and the magnetic separation and elution were performed the next day. Total EVs (small and large) were always isolated from plasma.

Human heart biopsies were obtained at the University of California San Diego, Cardiology Division (Institutional Review Board approval #181206). Subjects gave their informed consent for use of their explanted cardiac tissues for research and that our study adheres to the principles of the declaration of Helsinki.

### EV uptake assay

Freshly isolated large EVs (10 μg) from WT or *Rab7*^*−/−*^ MEF conditioned media were labeled with 5 μM CFSE (Invitrogen) in PBS for 20 min at room temperature and protected from ligh, according to the manufacturer’s instructions. A control solution was prepared with CFSE in PBS in the absence of EVs. The staining reaction was terminated by adding 5 ml cell culture medium plus 10% exosome depleted FBS. The CFSE-labeled large EVs were washed in PBS, centrifuged at 10,000 × *g* for 50 min and the resuspended in 20 μL of RPMI1640 + 10% exosome depleted FBS. The CFSE-labeled large EVs were added to RAW 264.7 macrophages (3 × 10^4^ per 60 mm MatTek dish) (MATTEK). After 20–24 h incubation at 37 °C, LysoTracker Red (75 nM) (Invitrogen) was added to the cells. After 0.5–1 h incubation at 37 °C, cells were imaged using a Nikon Eclipse microscope. EV uptake rate was quantified as the number of CFSE-positive cells divided by total number of cells per image.

### Cell death assay

To assess the level of cell death, WT MEFs were incubated with 5 nM Bafilomycin A1 (Millipore-sigma) for 48 h or *Rab7*^*−/−*^ MEFs expressing control or Rab27 shRNA lentiviruses were treated with DMSO or 50 nM Bafilomycin A1 or Oligomycin + Antimycin A (10 μM + 10 μM) (Sigma-Aldrich) for 24 h. To assess viability after treatments, cells were incubated with Yo-Pro1 (1:1000; Life Technologies) and Hoechst 33342 (1:1000; Invitrogen) for 15 min at 37 °C before imaging. Percent cell death was determined after treatments by dividing the number of Yo-Pro1-positive cells by total number of Hoechst 33342-positive cells as described^58^.

### Analysis of cardiac macrophages by flow cytometry

An adult mouse heart was excised, cannulated, and perfused with Liberase DH (26U/ml, Roche) in buffer containing 110 mM NaCl, 4.7 mM KCl, 0.6 mM NaHPO_4_, 0.6 mM KH_2_PO_4_, 1.25 mM MgSO_4_, 10 mM KHCO_3_, 12 mM NaHCO_3_, 5.5 mM glucose, 30 mM Taurine and 10 mM HEPES (pH 7.4). After digestion, ventricular tissue was gently teased into small pieces to dissociate loose cells. After about 30 min of sedimentation, the supernatant was centrifuged, and the cell pellet resuspended in staining buffer (BioLegend). Cells were seeded in 96-well plate, incubated with antibodies against CD45 (BioLegend, #14-0681-82, Dilution: 0.25 µg per million cells in 200 µl volume), CD11b (M1/70) (Tonbo Bioscience, #50-0112, Dilution: 0.25 µg per million cells in 200 µl volume) and F4/80 (Invitrogen, #17-4801-82, Dilution: 2 µg per million cells in 200 µl volume), and then analyzed using a Guava benchtop mini-flow cytometer (EMD Millipore). Data were quantified using FlowJo software (Version 10.8.1).

### Quantitative PCR

RNA was extracted from hearts using RNeasy Fibrous Tissue Mini Kit (Qiagen) and cDNA synthesized using the QuantiTect Reverse Transcription Kit (Qiagen). TaqMan primers *IL-6* (Mm00446190)*, Tnfα* (Mm00443258_m1)*, Il-1b* (Mm00434228_m1)*, Pink1 (*Mm00550827_m1), *Ppargc1a* (*PGC-1α* Mm01208835_m1), and *Tfam* (Mm00447485_m1)*, Rn18s* (Mm03928990_g1) were obtained from Thermo Fisher Scientific, while Nrf-1 (Forward: tggtccagagagtgcttgtg, Reverse: ttcctgggaagggagaagat) and Nrf-2 (Forward: ctcgctggaaaaagaagtgg, Reverse: ccgtccaggagttcagagag) custom primers were ordered from IDT. The TaqMan Universal Master Mix II (#4440040) was purchased from Applied Biosystems. The relative amounts of mRNA were normalized to *Rn18s* and the fold change of gene expression was calculated using the 2(−ΔΔCt) method. To measure mitochondrial DNA copy number in large EVs, genomic DNA was extracted using the GenElute Mammalian Genomic DNA Miniprep Kit (Sigma). *Rn18s* was used as a control for nuclear DNA content, and *cytochrome (Cyt) b* and *D-loop* were used for mtDNA quantitation as previously described^59^.

### Western blotting and antibodies

Proteins levels were analyzed by Western blotting using a standard protocol^59^. Antibodies used in this study were: anti-Rab7 (9367S, 1:1000), anti-Alix (2171S, 1:1000), anti-CD81 (10037S, 56039S, 1:1000), anti-Tom20 (42406S, 1:1000), anti-Rab4 (2167S, 1:1000), anti-Rab5 (3547S, 1:1000), anti-Rab9 (5118S, 1:1000), anti-Rab11 (5589S, 1:1000), anti-LC3A/B (4108S, 1:1000), anti-Rab27 (69295S, 1:1000), anti-Arl8b (56085 S, 1:1000), anti-Atg5 (12994S, 1:1000), anti-Atg7 (2631S, 1:1000) from Cell Signaling Technology; anti-SQSTM1/p62 (ab56416, 1:1000), anti-Tsg101 (ab83, 1:1000), anti-Calreticulin (ab2907, 1:1000) from Abcam; anti-Tim23 (11123-1-AP, 1:1000) from Proteintech, anti-CD63 (PA5-92370, 1:500) from Invitrogen; anti-dendra2 (TA150090, 1:1000) from OriGene; anti-GAPDH (GTX627408, 1:2000) from GeneTex; Anti-Ubiquitin (P4D1) (sc-8017, 1:1000) from Santa Cruz, MTCO1 (459600, 1:1000) from Thermo Fisher Scientific, and MnSOD (06-984, 1:1000) from Millipore. Goat anti-mouse HRP (31430; 1:5000) and Goat-anti-rabbit HRP (31460, 1:5000) secondary antibodies from Thermo Fisher Scientific.

### Proteinase K protection assay

Conditioned media from *Rab7*^*−/−*^ MEFs was collected from 12 T-182 flasks at 24 h and 48 h. Samples were centrifuged at 300 × *g* and 2000 × *g* each for 10 min to remove apoptotic bodies and other cellular debris. Large EVs were collected by centrifugation at 10,000 × *g* for 50 min and 45 μg of sample was subjected to treatment with 200 μg/mL Proteinase K (Clontech 740506) for 15 min on ice. Samples were incubated for 5 min with 5 mM PMSF to quench the Proteinase K reaction and EVs were collected by centrifugation at 10,000 × *g* for 50 min. The EV pellets were solubilized with triton x-100 lysis buffer and analyzed by Western blotting.

### Proteomics analysis

The proteomics analysis was done in collaboration with the Biomolecular/Proteomics Mass Spectrometry Facility at UCSD as described^60^. Briefly, proteins in isolated large and small EV fractions were separated by SDS–PAGE gel, subjected to in-gel tryptic digest and identified by ultra-high pressure liquid chromatography (UPLC) coupled with tandem mass spectroscopy (LC-MS/MS) using nano-spray ionization as described previously^59^. All plots were generated using the ggplot2 package in R and the Venn diagrams were generated using the VennDiagram package in R. Results were filtered by the number of unique peptide sequences detected. Proteins were used for analysis only if 2 or more unique peptides were detected. To account for low and missing values in the dataset, a background threshold value was calculated by taking the mean of all protein abundance values in the first quartile of the dataset, and this background value replaced missing values and all values lower than this threshold. Protein abundances were compared between large EV samples from WT and *Rab7*^*−/−*^ MEFs, and the −log10 adjusted *p*-values were plotted against the log2 fold changes. GO analysis was performed using the PANTHER statistical overrepresentation test (released 2022-02-02)^61^ on proteins significantly enriched in *Rab7*^*−/−*^ MEFs (*t*-test, *p* < 0.05). The whole *Mus musculus* genome was used as background. Pathway overrepresentation was calculated using Fisher’s Exact test with a Benjamini-Hoechberg multiple testing correction for a false discovery rate of 5%. The top hits from GO biological process complete and cellular component complete were plotted according to −log10 false discovery rate. To compare our dataset to previously existing proteins found in different types of extracellular vesicles, data from Vesiclepedia (Version 4.1, 2018-08-15) and ExoCarta (2015-07-29) were downloaded and compared to the set of proteins common to all WT and *Rab7*^*−/−*^ MEFs. The DeepLoc2.0 web tool was used to predict subcellular localization of proteins in WT vs. *Rab7*^*−/−*^ MEFs.

### Immunofluorescence microscopy

MEFs were plated on 35 mm glass bottom dishes (MatTek corporation) and treated with DMSO or Bafilomycin (50 nM) for 24 h. Cells were fixed with 4% PFA at 37 °C for 15 min and permeabilized with 0.2% Triton X- 100 at 37 °C for 30 min, followed by blocking in 5% normal goat serum^49^. Cells were incubated with primary and secondary antibodies in blocking solution overnight and 1 h, respectively. Primary antibodies: anti-Cytochrome *c* (BD Biosciences, 556432, 1:100); anti-CD81 (Cell Signaling Technology, 10037S, 1:100), anti-HSP60 (Cell Signaling Technology, 12165s, 1:100), anti-CD63 (Thermo Fisher Scientific, 10628D, 1:100). Secondary antibodies from Thermo Fisher Scientific: Goat anti-Rabbit IgG Alexa Fluor 488 (A-11034, 1:200), Goat anti-Mouse IgG Alexa Fluor 488 (A-11029, 1:200), Goat anti-Rabbit IgG Alexa Fluor 594 (A-11037, 1:200), Goat anti-Mouse IgG Alexa Fluor 594 (A-11032, 1:200). Nuclei were stained using Hoechst 33342 (Thermo Fisher Scientific). Z-stacks were captured using a Nikon Eclipse microscope equipped with DS-Qi2 camera and spinning-disk confocal system with a photometrics CMOS camera with Plan Apo λ 60x 1.4 NA (oil immersion) objective. Images prepared for publication were then processed for 3D deconvolution using Nikon NIS element software.

For live-cell imaging of CD81-GFP and mitochondria, MEFs were plated on 35 mm glass bottom MaTek dishes and transiently transfected with mPlum-mito-3 (Addgene plasmid #55988) and CD81-GFP (System Biosciences # CYTO124-PA-1). 24 h post-transfection, the media was changed, and images were captured using spinning-disk confocal microscope equipped with a Prime95B camera (Photometrics) and Plan Apo λ 100x 1.45 NA (oil immersion) objective. Z-stacks were acquired using a piezo Z stage (Mad City Labs) and images processed for 3D deconvolution using Nikon NIS element software. Time lapse experiments were performed with  the Purebox incubation system (Tokai Hit, Japan) to maintain  temperature, humidity, and CO2 levels.

To assess lysosomal pH, live MEFs were stained with 100 nM LysoTracker Red (Thermo Fisher Scientific, L7528) for 30 min at 37 °C. Nuclei were stained with 1 μg/ml Hoechst 33342 for 10 min prior fluorescence imaging. Z-stacks were captured using a Nikon Eclipse microscope equipped with DS-Qi2 camera with Plan Apo λ 60x 1.4NA (oil immersion) objective. Unprocessed and Z-stack images were used for integrated density calculation using ImageJ2 Version 2.3.0/1.53q software (NIH)/FIJI. The background was subtracted from the lysotracker red images (Ex 577 nm) followed by binary conversion and measured the integrated density.

For co-localization analysis, Z-stacks and unprocessed images were used for quantifications using green (Ex 488 nm) and red (Ex 594 nm) or far-red (Ex 647 nm) channels. Following the background subtraction from the images, the colocalization analysis was performed using the Just Another Colocalization Plugin (JACoP)^62^ in the FIJI software. The extent of colocalization was assessed using Mander’s overlap coefficient.

### 3D reconstruction

Images of frozen heart sections stained with anti-CD68 were captured using Nikon A1R confocal microscope with Plan apo λ 60x oil NA 1.40 objective and used for 3D reconstruction of cardiac mitochondria and macrophages using Imaris software version 10.0 (Bitplane AG, Switzerland). We used 0.4 μM surface detail and a threshold of 1.6 μm based on background subtraction. The source channels for the reconstruction of cardiomyocytes and cardiac macrophages were mito-dendra2-FITC and CD68-TRITC, respectively, and DAPI for nucleus.

### Transmission electron microscopy

Transmission electron microscopy was performed on heart sections from *Rab7*^*f/f*^ and *Rab7*^*f/f*^ *MCM* mice as described previously^59^. At D28 post-tamoxifen administration, hearts were fixed in 2.5% glutaraldehyde in 0.1 M cacodylate buffer, and post-fixed in 1% osmium tetroxide. EV pellets were fixed with 2.5% glutaraldehyde, 4% paraformaldehyde in 0.1 M sodium cacodylate buffer pH 7.2 for 30 min at RT, rinsed in the same buffer 3x 10 min, followed by post-fixation in reduced 1% osmium tetroxide in buffer for 40 min, rinsed in the same buffer 3x 10 min, stained with 1% uranyl acetate for 40 min, rinsed with ultra-pure water for 3x 10 min, dehydrated in a graded acetone series until absolute (3x) for 10 min each step and finally embedded in Epon resin. Ultrathin sections of 50 nm were obtained with a diamond knife (Diatome) using a ultramicrotome UC7 (Leica), transferred to 300 mesh copper grids and stained with Uranyless (EMS) for 5 min. Sections were observed in a Philips CM100 transmission electron microscope (FEI) operated at 100 kV. For negative staining, isolated large and small EV pellets were resuspended in PBS and a 10 μL drop of each sample were absorbed on Formvar/Carbon coated grids. After 3 times of washing using water drop, the grids were stained 1 min with 2% Uranyl Acetate in water. Representative images were captured using a JEOL JEM-1400Plus transmission electron microscope (JEOL, Peabody, MA) equipped with a Gatan OneView digital camera (Gatan, Pleasanton, CA).

For the correlated light and electron microscopy (CLEM), cells were plated on gridded MatTek dishes (MatTek Corporation) and transfected with CD81-GFP (System Biosciences # CYTO124-PA-1) and mPlum-mito-3 (Addgene plasmid #55988) using Fugene6 (Promega Inc.) according to the manufacturer’s instructions. The images were captured as previously described^6^.

### Statistical analysis

All experiments were performed in the laboratory with a minimum of three independently biological replicates. Data are expressed as mean ± SEM. Statistical significance between groups were determined using one-way ANOVA or two-way ANOVA tests with Tukey’s post-hoc test or Student’s *t*-test.

### Reporting summary

Further information on research design is available in the Nature Portfolio Reporting Summary linked to this article.

### Supplementary information

Supplementary Information

Description of Additional Supplementary Files

Supplementary Data 1

Supplementary Movie 1

Supplementary Movie 2

Reporting Summary

### Source data

Source Data

## Data Availability

All data associated with this study can be found in the paper, the Supplementary materials and Source data file. Research materials are available upon request. All proteomics data has been deposited in ProteomXchange via MassIVE partner repository (PXD037992 [ProteomeXchange Dataset PXD037992]). ExoCarta database can be found at http://www.exocarta.org. Vesiclepedia data base can be found at http://microvesicles.org. Source data are provided with this paper.
