# Supplementary material for: Mitochondria are secreted in extracellular vesicles when lysosomal function is impaired
Source: Nat Commun. 2023 Aug 18;14:5031. doi: 10.1038/s41467-023-40680-5 (PMC10439183; doi:10.1038/s41467-023-40680-5)
Supplement: Supplementary file 1 — Supplementary Information [file 41467_2023_40680_MOESM1_ESM.pdf]

Supplementary information for Mitochondria are Secreted in Extracellular Vesicles When Lysosomal Function is Impaired

Supplementary Figures 1-9

Supplementary Figure 1. EV release is increased in MEFs when lysosomal function is compromised.

Supplementary Figure 2. EV release is increased in hearts when lysosomal function is compromised.

Supplementary Figure 3. EV release is increased in Rab7-deficient cells.

Supplementary Figure 4. Endosomal proteins are increased in *Rab7*$^{-/-}$ MEFs.

Supplementary Figure 5. EV secretion can occur independently of autophagosome formation and mitophagy.

Supplementary Figure 6. Effect of Rab27a knockdown in *Rab7*-deficient cells.

Supplementary Figure 7. Characterization of *Rab7*$^{f/f}$ *MCM* mice.

Supplementary Figure 8. Characterization of EV release in hearts.

Supplementary Figure 9. Release of small EVs is unchanged in aged (24 months) or *Lamp2*-deficient hearts.

Supplementary Figure 1

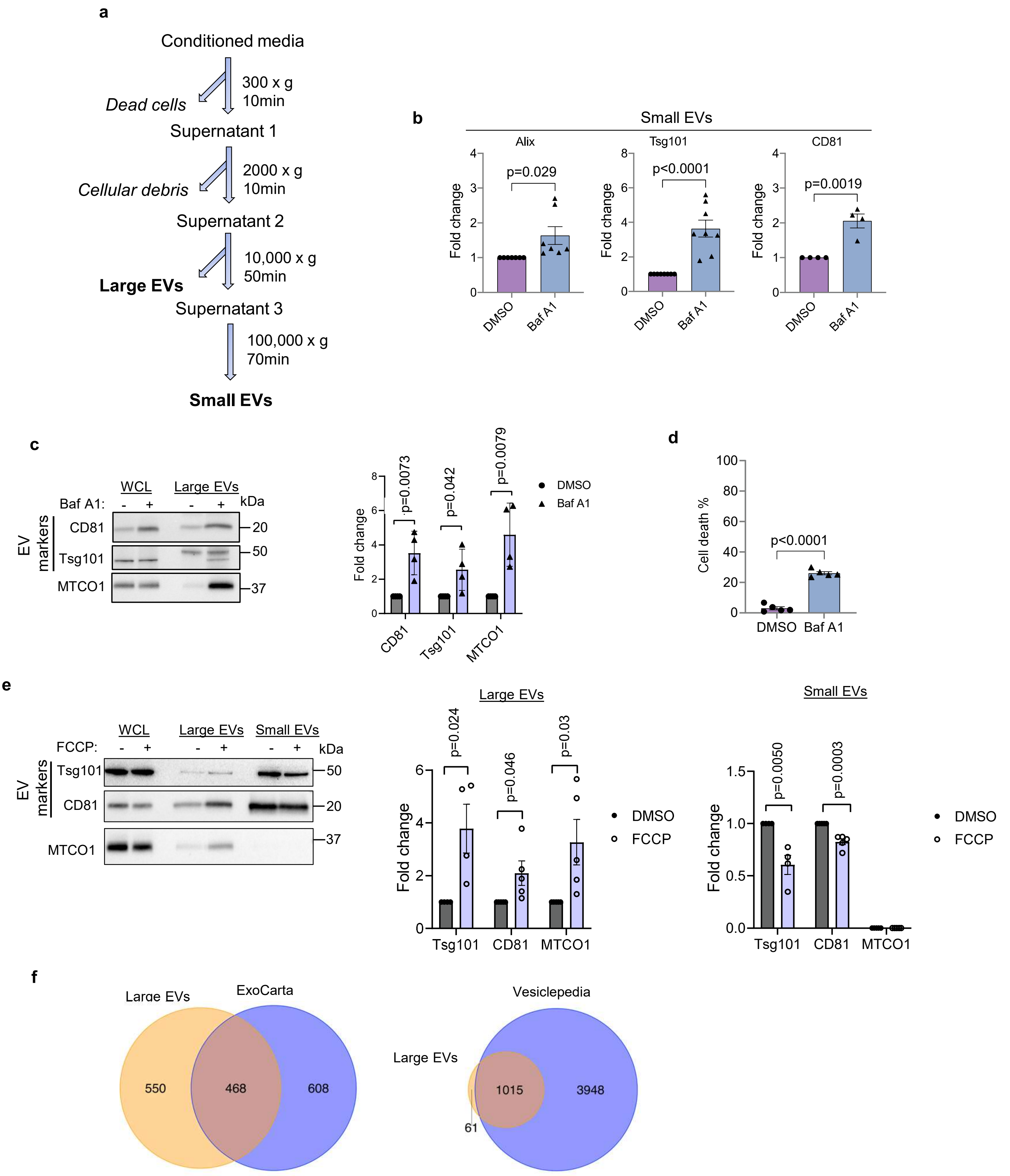

Supplementary Figure 1. EV release is increased in MEFs when lysosomal function is compromised. a. Scheme of EV isolation from conditioned media using differential centrifugation or immunoaffinity purification. The immunoaffinity purification method uses an antibody cocktail against tetraspanin surface markers CD9, CD63 and CD81. **b.** Quantification of proteins in small EV fraction from Fig. 1a (Alix n=7; Tsg101 n=8, CD81 n=4 biologically independent experiments). **c.** Representative Western blot and quantification of large EVs isolated using affinity capture (n=4 biologically independent experiments). **d.** Evaluation of cell death in MEFs after 48 h of Baf A1 treatment (5 nM) (n=5 biologically independent experiments). **e.** Representative Western blot of proteins in WCL and EVs secreted by WT MEFs after treatment with FCCP (10 μM) for 24 h. Quantification of large and small EVs (Tsg101 n=4; CD81 n=5, MTCO1 n=5 biologically independent experiments)). **f.** Venn diagrams showing the overlap of proteins identified in large EVs with those reported in Vesiclepedia and ExoCarta databases. Data are mean±SEM. P values shown are by two-sided Student's t-test. Source data are provided as a Source Data file.

Supplementary Figure 2

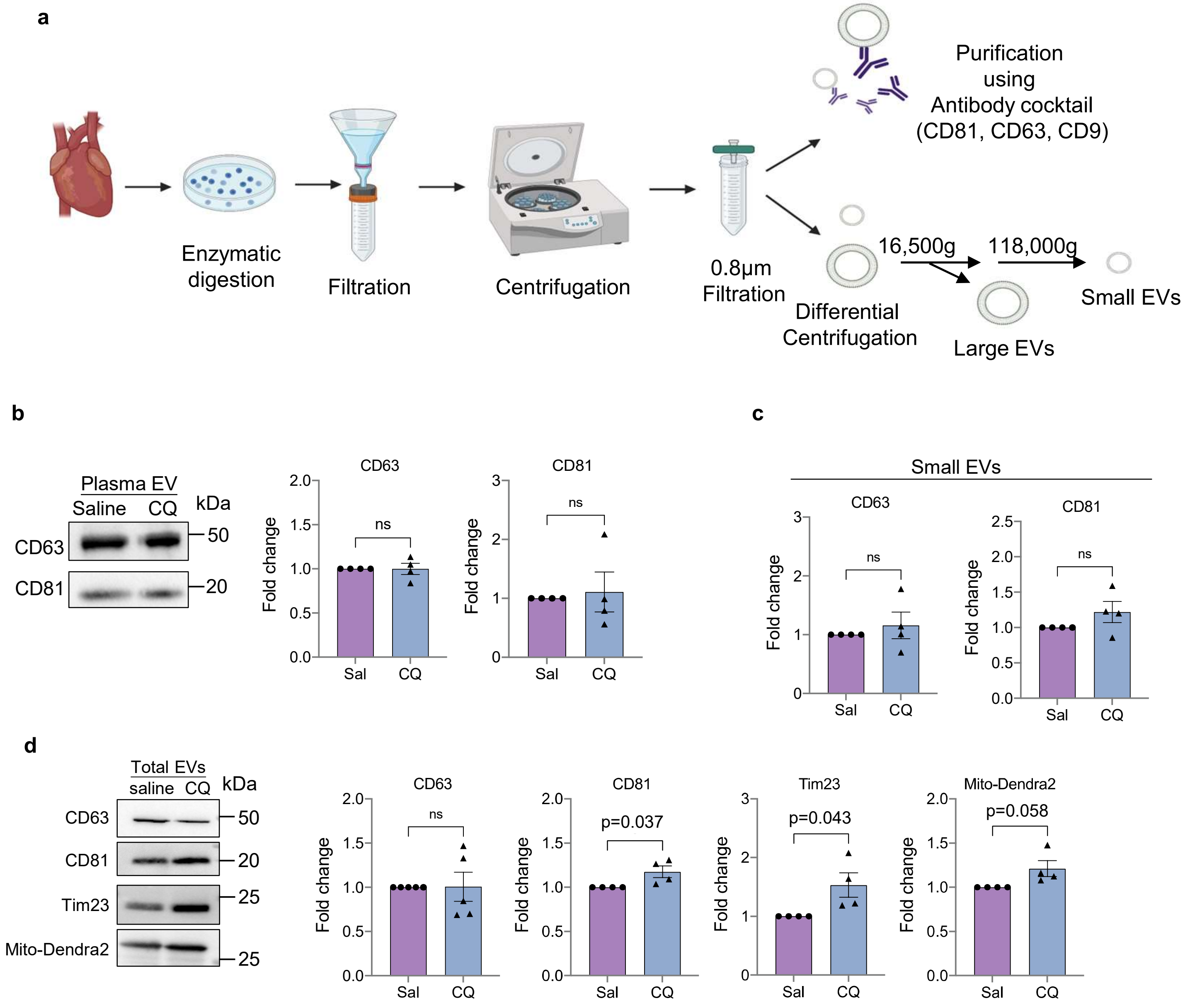

Supplementary Figure 2. EV release is increased in hearts when lysosomal function is compromised. a. Scheme of EV isolation from heart tissue (Created using BioRender.com). **b.** Western blot analysis and quantification of total EVs isolated from mouse plasma (n=4 biologically independent experiments). **c.** Quantification of proteins in small EV fraction from Fig. 1e (n=4 biologically independent experiments). **d.** Western blot analysis and quantification of CD63, CD81, Tim23, and Mito-Dendra2 protein levels in EV fractions isolated from heart tissue using immunoaffinity capture (n=4 biologically independent experiments). Data are mean±SEM. ns = not significant. P values shown are by two-sided Student's t-test. Source data are provided as a Source Data file.

Supplementary Figure 3

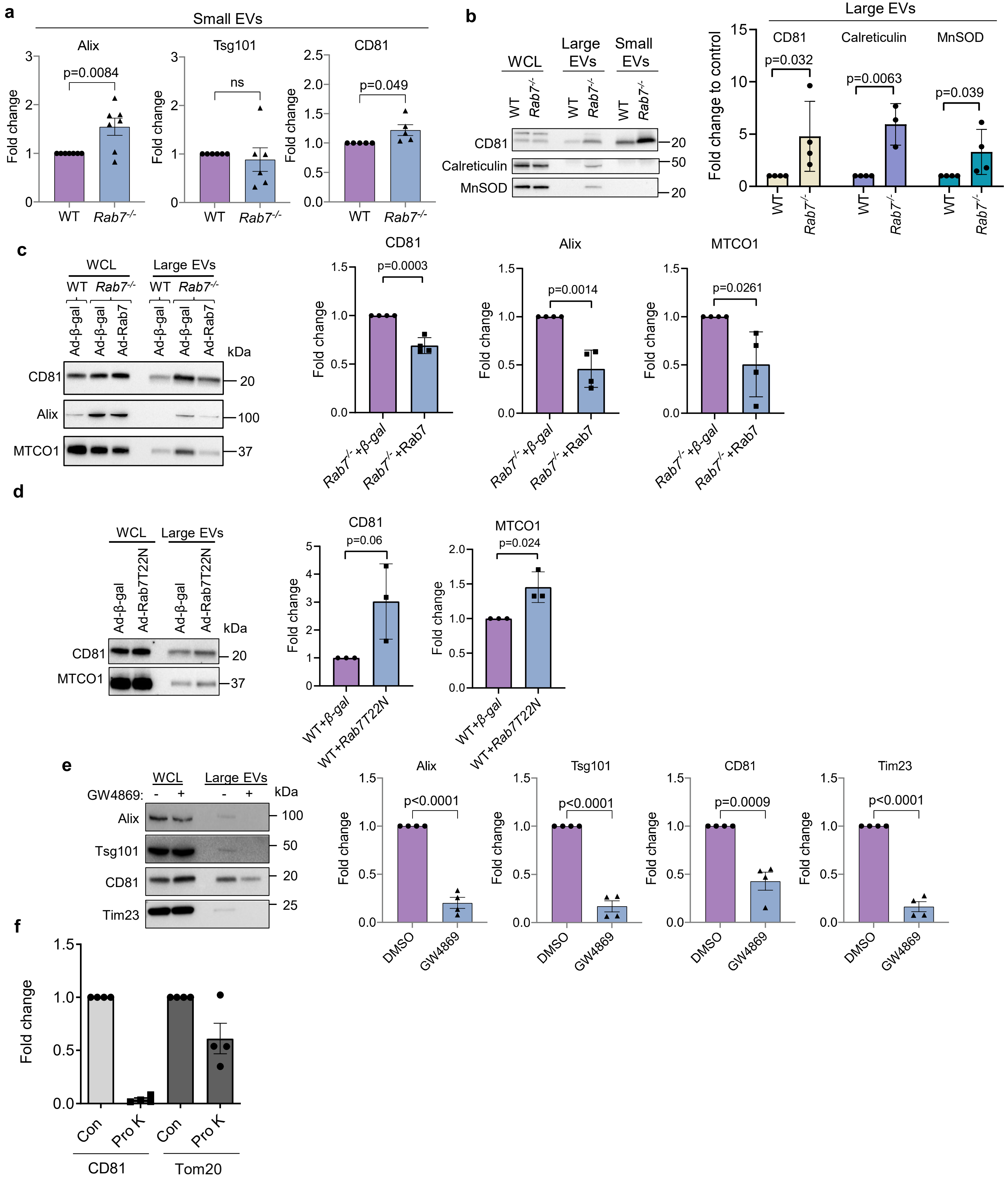

Supplementary Figure 3. EV release is increased in Rab7-deficient cells. a. Quantification of proteins in small EV fraction from Fig. 2a (Alix n=7; Tsg101 n=6, CD81 n=5 biologically independent experiments). **b**. Representative Western blot and quantification of CD81 (EV marker, n=4 biologically independent experiments), Calreticulin (ER, n=3 biologically independent experiments) and MnSOD (mitochondria, n=4 biologically independent experiments) in whole cell lysates (WCL) and EV fractions from WT and *Rab7*$^{-/-}$ MEFs. **c.** Representative Western blot and quantification of proteins in WCL and large EVs (n=4 biologically independent experiments). Cells were infected with Ad-β-gal or Ad-Rab7 for 24h. **d.** Representative Western blot and quantification of proteins in WCL and large EVs (n=4 biologically independent experiments). Cells were infected with Ad-β-gal or Ad-Rab7T22N for 36h. **e**. Representative Western blot of proteins in whole cell lysates (WCL) and large EV fraction from *Rab7*$^{-/-}$ MEFs after treatment with GW4869 (2.5μM, 48hr). Quantification of proteins in the large EV fraction (n=4 biologically independent experiments). **f**. Quantification of CD81 and Tom20 levels in large EVs after proteinase K digestion (n=4 biologically independent experiments). Data are mean±SEM. P values shown are by two-sided Student's t-test. Source data are provided as a Source Data file.

Supplementary Figure 4

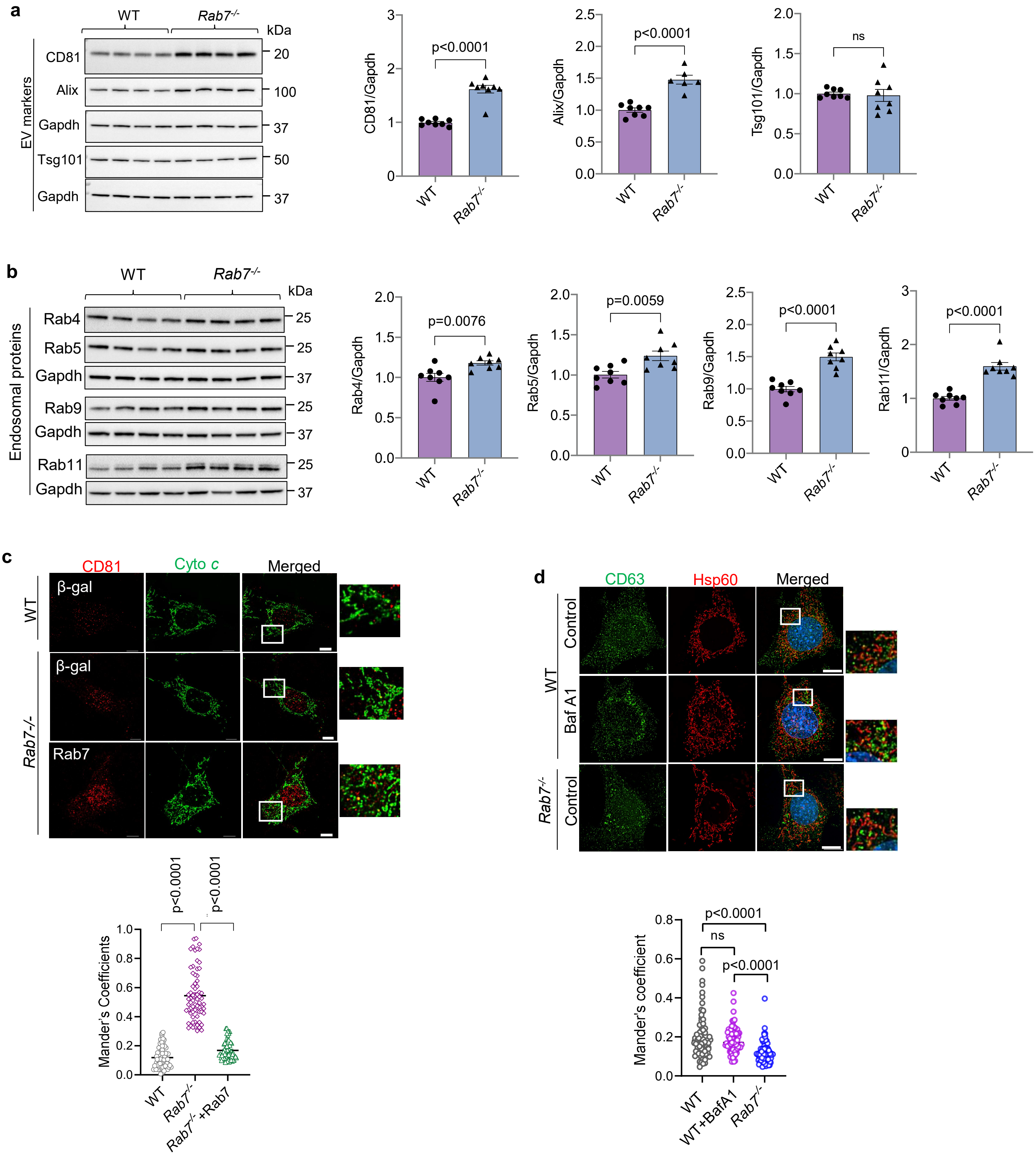

Supplementary Figure 4. Endosomal proteins are increased in *Rab7$^{-/-}$* MEFs. a. Representative western blot and quantification of various EV proteins in WT and *Rab7$^{-/-}$* MEFs (CD81/Gapdh and Tsg101/Gapdh: WT and *Rab7$^{-/-}$* (n=8 biologically independent experiments); Alix/Gapdh: WT (n=8 biologically independent experiments) and *Rab7$^{-/-}$* (n=6 biologically independent experiments). **b**. Representative Western blot and quantification of endosomal proteins in WT and *Rab7$^{-/-}$* MEFs (n=8 biologically independent experiments). **c.** Co-localization between CD81 (red) and Cytochrome *c* (green) in WT and *Rab7$^{-/-}$* infected with the indicated adenovirus for 24 h. Scale bar = 20µm. Mander's overlap coefficient (n=3 independent experiments with a total of 90 cells). **d.** Co-localization analysis between CD63 (green) and Hsp60 (red, mitochondria) in WT +/- Baf A1 (50nM for 24hr) and *Rab7$^{-/-}$* MEFs. Scale bar = 20µm. Mander's overlap coefficient (n=3 independent experiments with a total of 90 cells). Data are mean±SEM. ns = not significant. P values shown are by two-sided Student's t-test (**a**, **b**) or ANOVA with Tukey's post-hoc testing (**c, d**). Source data are provided as a Source Data file.

Supplementary Figure 5

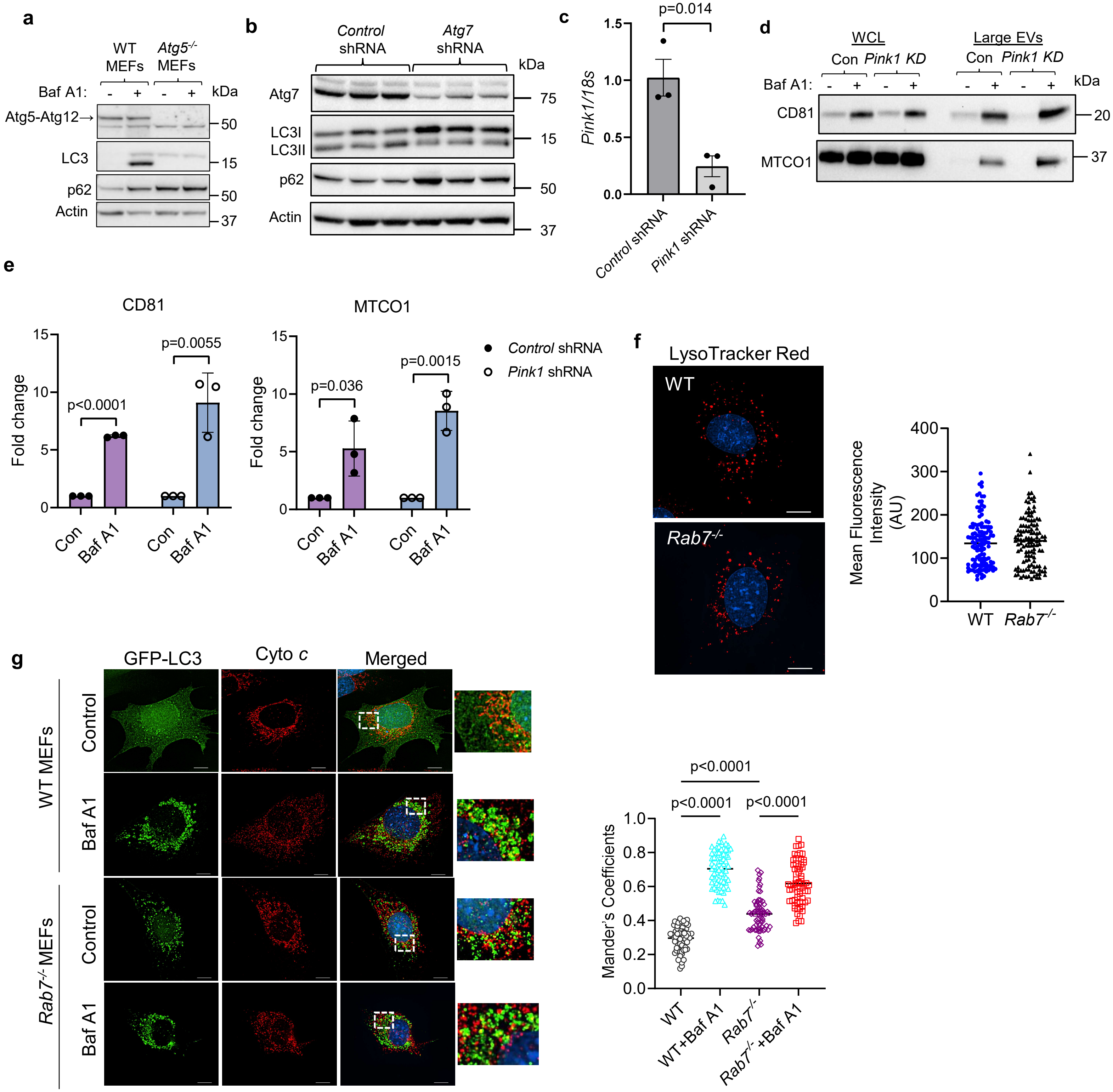

Supplementary Figure 5. EV secretion can occur independently of autophagosome formation and mitophagy. a. Western blotting for Atg5, LC3, and p62 protein levels in WT and *Atg5*$^{-/-}$ MEFs. Atg5 exists in a complex with Atg12 in WT MEFs (representative from n=3 independent experiments). **b.** Quantification of proteins after Atg7 knockdown (n=3 biologically independent experiments). **c.** qPCR analysis for Pink1 transcript levels in MEFs infected with *control* or *Pink1* shRNA lentiviral particles (2 MOI, n=3). **d.** Representative Western blots of whole cell lysate (WCL) and extracellular vesicle fractions for CD81 and MTCO1. The EVs were obtained by differential centrifugation of conditioned media from MEFs collected over 48 h +/- Baf A1 (50nM for 24hr). **e** Quantification of proteins in large EVs (n=3 biologically independent experiments). **f.** LysoTracker Red staining of cells and quantification of fluorescence intensity (n=3 independent experiments with a total of 90 cells scored). **g.** Representative images of co-localization between GFP-LC3 (green) and Cytochrome *c* (red) in WT and *Rab7*$^{-/-}$ MEFs +/- Baf A1 (50nM for 24hr). Mander's correlation coefficient (n=3 independent experiments with a total of 90 cells scored). All scale bars = 20 μm. Data are mean±SEM. ****p<0.0001. P values shown are by two-sided Student's t-test (**c**, **e**) or ANOVA with Tukey's post-hoc testing (**g**). Source data are provided as a Source Data file.

Supplementary Figure 6.

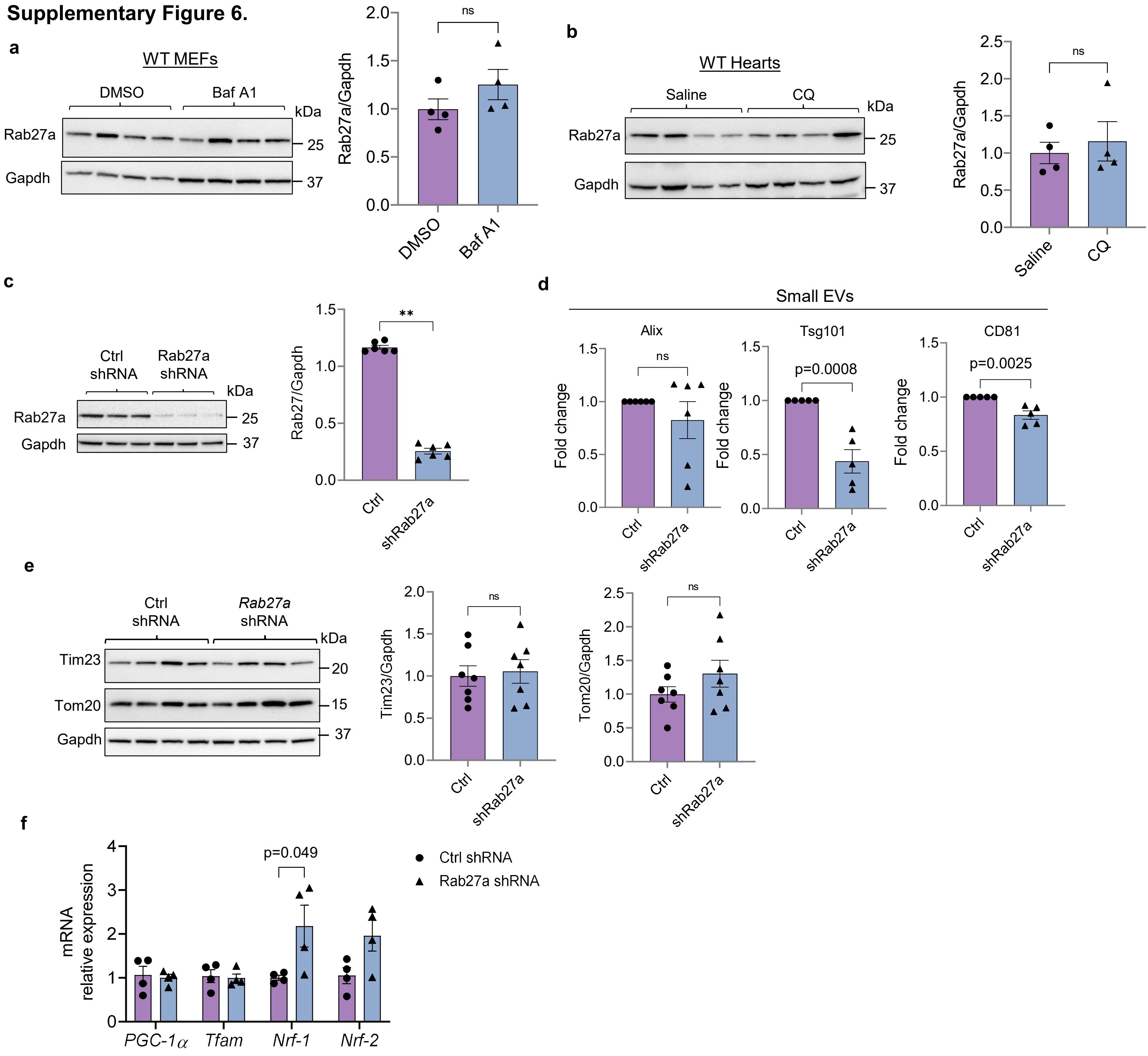

Supplementary Figure 6. Effect of Rab27a knockdown in *Rab7*-deficient cells. a. Western blot for Rab27 levels in WT MEFs after treatment with BafA1 (5nM, 24h, n=4 biologically independent experiments). **b.** Western blot for Rab27 in WT mouse hearts 24 h after administration of chloroquine (CQ) (n=4 biologically independent animals). **c.** Confirmation of Rab27 knockdown in *Rab7*$^{-/-}$ MEFs. Representative western blot and quantification of Rab27 protein levels in WT and *Rab7*$^{-/-}$ MEFs after infection with control or Rab27a shRNA lentivirus (n=6 biologically independent experiments). **d.** Quantification of proteins in small EV fractions from Fig. 5e (Alix n=6; Tsg101, CD81 n=5 biologically independent experiments). **e.** Representative Western blots and quantification of mitochondrial proteins in MEFs after Rab27 knockdown (n=7 biologically independent experiments). **f.** Analysis of mRNA levels by qPCR in WT MEFs (n=4 biologically independent experiments). Data are mean±SEM. All sample numbers represents biologically independent experiments. ns = not significant. P values shown are by two-sided Student's t-test. Source data are provided as a Source Data file.

Supplementary Figure 7

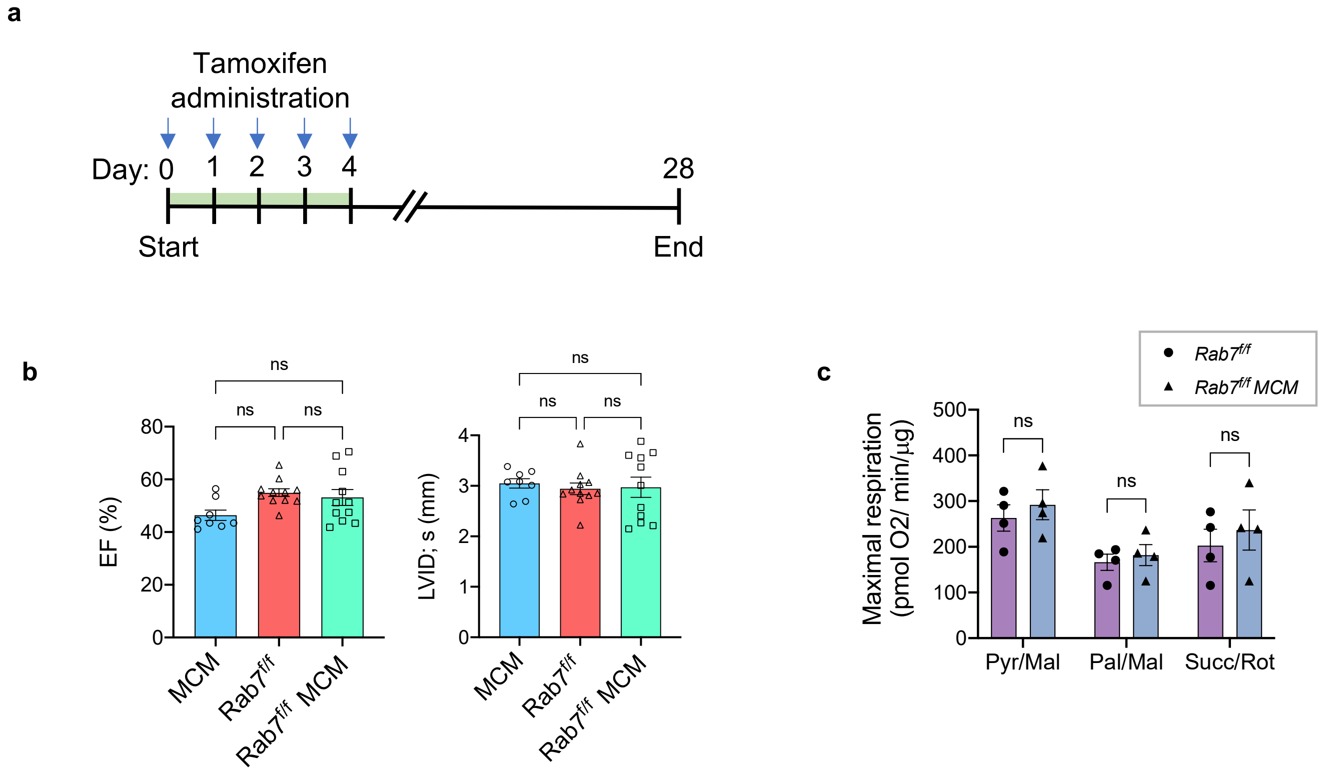

Supplementary Figure 7. Characterization of *Rab7$^{f/f}$ MCM* mice. a. Mice were injected with tamoxifen (40 mg/kg) for 5 days and all phenotyping conducted at 28 days post-tamoxifen treatment. **b.** Echocardiographic analysis of ventricular function and structure 28 days post-tamoxifen treatment. Percent (%) ejection fraction (EF) and left ventricular internal dimension in systole (LVED; s) . *MCM* (n=8 biologically independent animals), *Rab7$^{f/f}$* (n=11 biologically independent animals), *Rab7$^{f/f}$ MCM* (n=11 biologically independent animals). **c**. Assessment of mitochondrial respiration using isolated mitochondria from *Rab7$^{f/f}$ and Rab7$^{f/f}$ MCM* hearts show no differences in maximal respiration rates (FCCP uncoupled) with substrates for complex I (pyruvate/malate and palmitoyl carnitine/malate) or II (succinate/rotenone) (*n* = 4 biologically independent samples). Data are mean±SEM. ns = not significant. Statistical test performed by ANOVA with Tukey's post-hoc testing (**b**) or two-sided Student's t-test (**c**). Source data are provided as a Source Data file.

Supplementary Figure 8

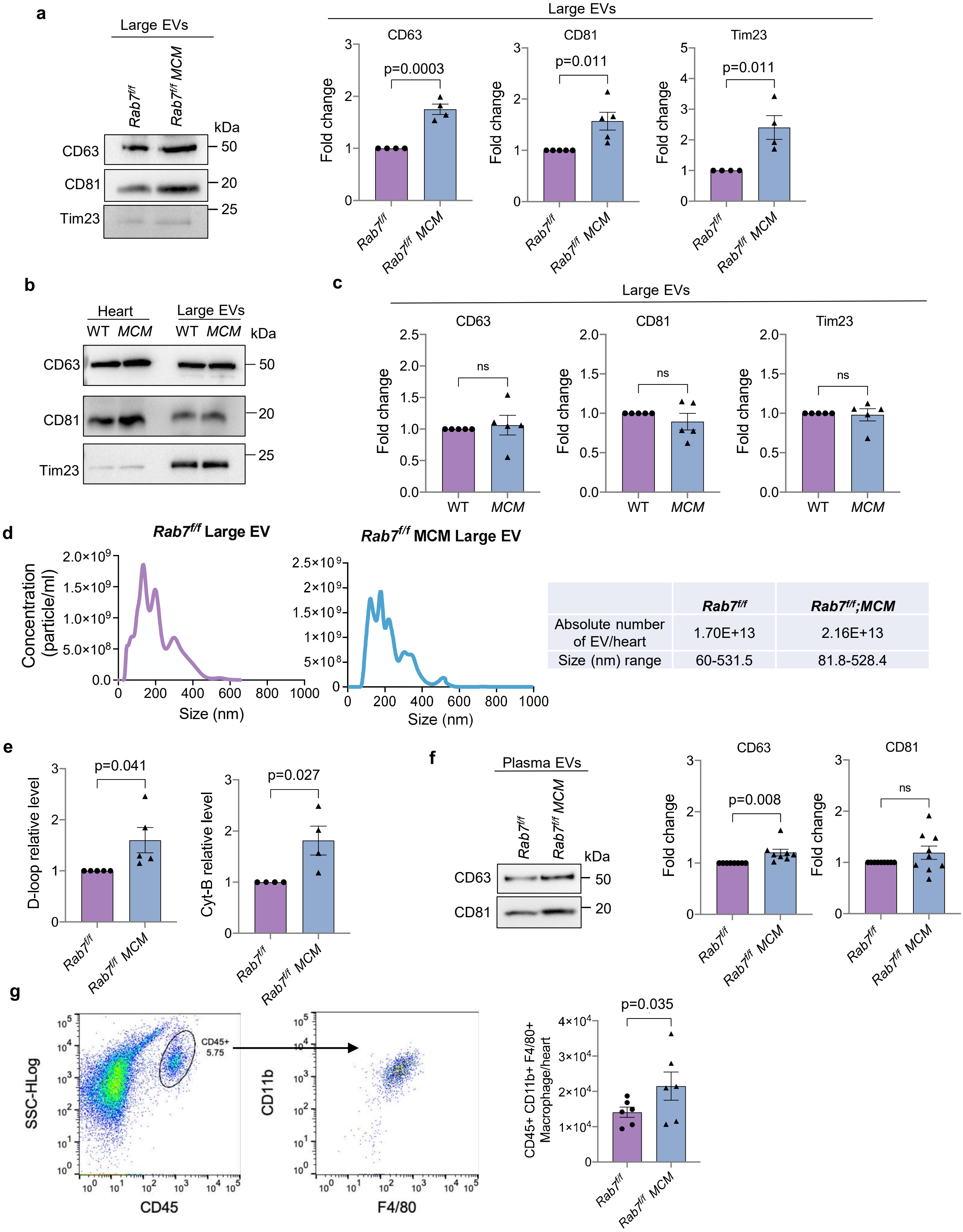

Supplementary Figure 8. Characterization of EV release in hearts. **a.** Representative Western blots and quantification of proteins in large EV fractions isolated from hearts using immunoaffinity purification (CD63, Tim23: *Rab7*$^{f/f}$ and *Rab7*$^{f/f}$ *MCM* (n=4 biologically independent animals); CD81: *Rab7*$^{f/f}$ and *Rab7*$^{f/f}$ *MCM* (n=5 biologically independent animals)). **b.** Representative Western blots of protein levels in large EV fractions from WT and *MCM* heart tissue at D28 post-tamoxifen injection. **c.** Quantification of proteins in large EV fractions (WT and *MCM,* n=5 biologically independent animals). **d.** Nano particle tracking analysis (NTA) to assess size distribution of large EVs isolated from *Rab7*$^{f/f}$ and *Rab7*$^{f/f}$ *MCM* heart tissue. **e.** Mitochondrial DNA (mtDNA) content in large EVs preparations (D-loop n=5, Cyt B n=4 biologically independent animals). **f.** Representative Western blots and quantification of proteins in total (large and small) EV fractions isolated from plasma using immunoaffinity purification (CD63 n=8, CD81 n=9 biologically independent animals). **g.** Gating strategy for the macrophage content analysis and quantification of cardiac macrophage content in *Rab7*$^{f/f}$ and *Rab7*$^{f/f}$ *MCM* hearts analyzed by flow cytometry (n=6 biologically independent animals). Data are mean±SEM. ns = not significant. P values shown are by two-sided Student's t-test. Source data are provided as a Source Data file.

Supplementary Figure 9

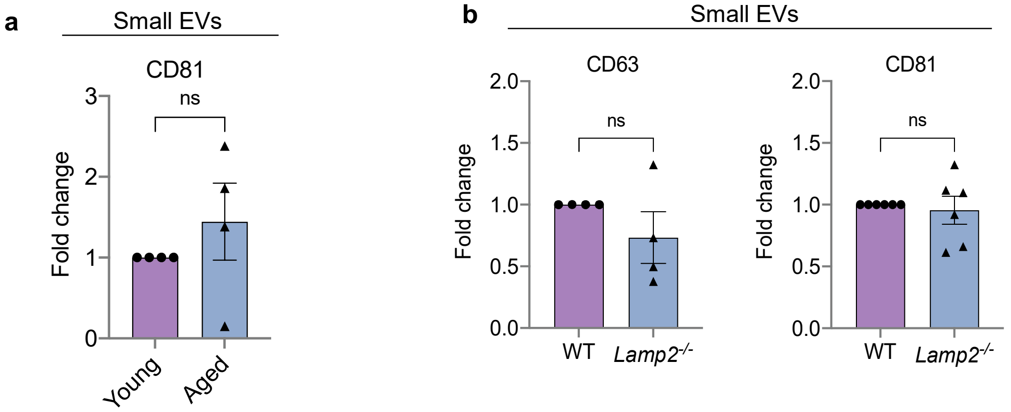

Supplementary Figure 9. Release of small EVs is unchanged in aged (24 months) or *Lamp2*-deficient hearts. a. Quantification of proteins in small EV fractions from Fig. 8a (n=4 biologically independent animals). **b**. Quantification of CD63 (n=4 biologically independent animals) and CD81 (n=6 biologically independent animals) proteins in small EV fractions from Fig. 8d. Data are mean±SEM. ns=not significant. Statistical analysis performed by two-sided Student's t-test. Source data are provided as a Source Data file.
