# Supplementary material for: Mitochondria are secreted in extracellular vesicles when lysosomal function is impaired
Source: Nat Commun. 2023 Aug 18;14:5031. doi: 10.1038/s41467-023-40680-5 (PMC10439183; doi:10.1038/s41467-023-40680-5)
Supplement: Supplementary file 2 — Description of Additional Supplementary Files [file 41467_2023_40680_MOESM2_ESM.pdf]

Description of Additional Supplementary Files

Supplementary Data 1: Related to figures 1 and 2. Proteomics data sets for Venn diagram in figure 1c comparing small and large EVs, GO enrichment analysis of unique proteins identified in large EV fractions (figure 1d), Volcano plot of proteins identified in large EV fractions from WT and *Rab7*$^{-/-}$ MEFs (figure 2h), and GO enrichment analysis of EV proteins isolated from *Rab7*$^{-/-}$ MEFs relative to wild type cells (figure 2i).

Supplementary Movie 1. Related to figure 3c. Time-lapse imaging of a *Rab7*$^{-/-}$ MEF overexpressing CD81-GFP and mPlum-mito3. MitoPlum related to Figure 3. Images were captured every 20 s (0-160 s). Images demonstrate recruitment of CD81-GFP-positive vesicles (green) to mitochondria (red).

Supplementary Movie 2. Related to figure 7j. Reconstruction and 3D view of a macrophage labeled with anti-CD68 containing Mito-Dendra2-positive mitochondria (green).
