# Supplementary material for: Mitochondria are secreted in extracellular vesicles when lysosomal function is impaired
Source: Nat Commun. 2023 Aug 18;14:5031. doi: 10.1038/s41467-023-40680-5 (PMC10439183; doi:10.1038/s41467-023-40680-5)
Supplement: Supplementary file 6 — Reporting Summary [file 41467_2023_40680_MOESM6_ESM.pdf]

nature portfolio

Corresponding author(s): Asa B. Gustafsson

Last updated by author(s): Jul 25, 2023

Reporting Summary

Nature Portfolio wishes to improve the reproducibility of the work that we publish. This form provides structure for consistency and transparency in reporting. For further information on Nature Portfolio policies, see our Editorial Policies and the Editorial Policy Checklist.

Statistics

For all statistical analyses, confirm that the following items are present in the figure legend, table legend, main text, or Methods section.

n/a	Confirmed	
☐	☒	The exact sample size (*n*) for each experimental group/condition, given as a discrete number and unit of measurement
☐	☒	A statement on whether measurements were taken from distinct samples or whether the same sample was measured repeatedly
☐	☒	The statistical test(s) used AND whether they are one- or two-sided<br>*Only common tests should be described solely by name; describe more complex techniques in the Methods section.*
☒	☐	A description of all covariates tested
☐	☒	A description of any assumptions or corrections, such as tests of normality and adjustment for multiple comparisons
☐	☒	A full description of the statistical parameters including central tendency (e.g. means) or other basic estimates (e.g. regression coefficient) AND variation (e.g. standard deviation) or associated estimates of uncertainty (e.g. confidence intervals)
☐	☒	For null hypothesis testing, the test statistic (e.g. *F*, *t*, *r*) with confidence intervals, effect sizes, degrees of freedom and *P* value noted<br>*Give P values as exact values whenever suitable.*
☒	☐	For Bayesian analysis, information on the choice of priors and Markov chain Monte Carlo settings
☒	☐	For hierarchical and complex designs, identification of the appropriate level for tests and full reporting of outcomes
☒	☐	Estimates of effect sizes (e.g. Cohen's *d*, Pearson's *r*), indicating how they were calculated

Our web collection on statistics for biologists contains articles on many of the points above.

Software and code

Policy information about availability of computer code

Data collection	NIS Elements Imaging Software (Nikon) Version 5.21.02 for immunofluorescence microscopy<br>SoftMax Pro Software (Molecular Devices) Version 5.4 for Bradford protein assay<br>Image Lab Beta 2 (Bio Rad) Version 3.0.1 for Western blot visualization<br>CFX Manager 2.0 Software (Bio Rad) Version 2.0.885.0923 for qPCR.<br>Vevo Lab (Visual Sonics) Version 3.2.0 for echocardiography.
Data analysis	Image Lab Beta 2 (Bio Rad) Version 3.0.1 or Quantity one (Bio Rad) Version 4.6.9 for Western blot band densitometry.<br>All statistical analyses and P values were obtained using the GraphPad Prism software version 7.0 (GraphPad Software, Inc. USA).<br>NIS Elements Imaging Software (Nikon) Version 5.21.02 for immunofluorescence analysis<br>ImageJ2 Version 2.3.0/1.53q software (NIH)/FIJI with Just Another Colocalization Plugin for analysis of fluorescent images.<br>Imaris software version 10.0 (Bitplane AG, Switzerland) for 3D reconstruction<br>Vevo 3100 software (Visual sonics) was used for echocardiography<br>RStudio Version 2022.12.0+353 (2022.12.0+353) (RStudio, Inc., USA)<br>FlowJo Version 10.8.1

For manuscripts utilizing custom algorithms or software that are central to the research but not yet described in published literature, software must be made available to editors and reviewers. We strongly encourage code deposition in a community repository (e.g. GitHub). See the Nature Portfolio guidelines for submitting code & software for further information.

Data

Policy information about availability of data

All manuscripts must include a data availability statement. This statement should provide the following information, where applicable:

- Accession codes, unique identifiers, or web links for publicly available datasets
- A description of any restrictions on data availability
- For clinical datasets or third party data, please ensure that the statement adheres to our policy

All data associated with this study can be found in the paper, the Supplementary materials and Source data file. Uncropped blots of all westerns have also been provided. The proteomic datasets generated in this study have been deposited to the ProteomeXchange Consortium (https://www.proteomexchange.org/) via the MassIVE partner repository (Project accession: PXD037992). ExoCarta database can be found at http://www.exocarta.org/. Vesiclepedia data base can be found at http://microvesicles.org/.

Human research participants

Policy information about studies involving human research participants and Sex and Gender in Research.

Reporting on sex and gender	1 Female and 3 Males are included in this project
Population characteristics	Danon disease patients' information: 17-year-old male diagnosed with Danon Disease and had a total artificial heart. 39-year-old female with Danon disease and end-stage heart failure; Control patients information: 34-year-old male organ donor without significant past medical history who died from blunt head trauma and had no cardiovascular abnormalities on echocardiogram; 17-year-old male without significant medical history who died from drug overdose and had no cardiovascular abnormalities on echocardiogram.
Recruitment	Subjects gave their informed consent for use of their explanted cardiac tissues for research and that our study adheres to the principles of the declaration of Helsinki.
Ethics oversight	University of California San Diego, Cardiology Department (Institutional Review Board approval #181206)

Note that full information on the approval of the study protocol must also be provided in the manuscript.

Field-specific reporting

Please select the one below that is the best fit for your research. If you are not sure, read the appropriate sections before making your selection.

☒ Life sciences ☐ Behavioural & social sciences ☐ Ecological, evolutionary & environmental sciences

For a reference copy of the document with all sections, see nature.com/documents/nr-reporting-summary-flat.pdf

Life sciences study design

All studies must disclose on these points even when the disclosure is negative.

Sample size	No statistical method was used to predetermine the sample size. The sample sizes were chosen to assure statistical differences and reproducibility of the results, and based on the study designs in previous studies in our laboratory using similar methods (PMID:36719945,PMID:32717194).
Data exclusions	Data exclusion criteria was predetermined. Scientific reasons to exclude data included very low transfection efficiency (<25%) or if cell viability was affected in control conditions.
Replication	All replication attempts were successful. Experiments were independently repeated at least three times with similar results.
Randomization	Animals were allocated into experimental groups randomly and litter mates were used as controls whenever possible.
Blinding	The investigators were not blinded to experimental group assignments in animal, cellular, and biochemical studies because the experiments required various treatments (shRNA, drug treatments) and/or genotyping by the investigators. Only the echocardiographic data were collected in a blinded manner by an investigator that was not involved in maintaining/genotyping mice for the study.

Reporting for specific materials, systems and methods

We require information from authors about some types of materials, experimental systems and methods used in many studies. Here, indicate whether each material, system or method listed is relevant to your study. If you are not sure if a list item applies to your research, read the appropriate section before selecting a response.

Materials & experimental systems

n/a	Involved in the study
☐	☒ Antibodies
☐	☒ Eukaryotic cell lines
☒	☐ Palaeontology and archaeology
☐	☒ Animals and other organisms
☒	☐ Clinical data
☒	☐ Dual use research of concern

Methods

n/a	Involved in the study
☒	☐ ChIP-seq
☐	☒ Flow cytometry
☒	☐ MRI-based neuroimaging

Antibodies

Antibodies used

Antibodies used in WB were:
anti-Rab7 Rabbit mAb supplier: Cell Signaling Technology, catalog number: 9367S, Dilution: 1:1000
anti-Alix Mouse mAb supplier: Cell Signaling Technology, catalog number: 2171S, Dilution: 1:1000
anti-CD81 Rabbit mAb supplier: Cell Signaling Technology, catalog number: 10037S, Dilution: 1:1000
anti-CD81 Rabbit mAb supplier: Cell Signaling Technology, catalog number: 56039S, Dilution: 1:1000
anti-Tom20 Rabbit mAb supplier: Cell Signaling Technology, catalog number: 42406S, Dilution: 1:1000
anti-Rab4 Rabbit pAb supplier: Cell Signaling Technology, catalog number: 2167S, Dilution: 1:1000
anti-Rab5 Rabbit mAb supplier: Cell Signaling Technology, catalog number: 3547S, Dilution: 1:1000
anti-Rab9A Rabbit mAb supplier: Cell Signaling Technology, catalog number: 5118S, Dilution: 1:1000
anti-Rab11 Rabbit mAb supplier: Cell Signaling Technology, catalog number: 5589S, Dilution: 1:1000
anti-LC3A/B Rabbit pAb supplier: Cell Signaling Technology, catalog number: 4108S, Dilution: 1:1000
anti-Rab27A Rabbit mAb supplier: Cell Signaling Technology, catalog number: 69295S, Dilution: 1:1000
anti-Arl8b Rabbit pAb supplier: Cell Signaling Technology, catalog number: 56085S, Dilution: 1:1000
anti-Atg5 Rabbit mAb supplier: Cell Signaling Technology, catalog number: 12994S, Dilution: 1:1000
anti-Atg7 Rabbit pAb supplier: Cell Signaling Technology, catalog number: 2631S, Dilution: 1:1000
anti-SQSTM1/p62 Mouse mAb supplier: abcam, catalog number: ab5641, Dilution: 1:1000
anti-Tsg101, Mouse mAb supplier: abcam, catalog number: ab83, Dilution: 1:1000
anti-Calreticulin Rabbit mAb supplier: abcam, catalog number: ab2907, Dilution: 1:1000
anti-Tim23 Rabbit pAb supplier: proteintech, catalog number: 11123-1-AP, Dilution: 1:1000
anti-CD63 Rabbit pAb supplier: Invitrogen, catalog number: PA5-92370, Dilution: 1:500
anti-dendra2 Rabbit pAb supplier: OriGene, catalog number: TA150090, Dilution: 1:1000
anti-GAPDH Mouse mAb supplier: GeneTex, catalog number: GTX627408, Dilution: 1:2000
anti-Ubiquitin (P4D1) Mouse mAb supplier: Santa Cruz, catalog number: sc-8017, Dilution: 1:1000
anti-MTCO1 Mouse mAb supplier: Thermo Fisher Scientific, catalog number: 459600, Dilution: 1:1000
anti-MnSOD Rabbit pAb supplier: Millipore Sigma, catalog number: 06-984, Dilution: 1:1000
Goat anti-mouse HRP secondary antibody supplier: Thermo Fisher Scientific, catalog number: 31430, Dilution: 1:5000
Goat-anti-rabbit HRP secondary antibody supplier: Thermo Fisher Scientific, catalog number: 31460, Dilution: 1:5000
For immunostaining:
anti-Cytochrome c Mouse 6H2.B4 mAb supplier: BD Biosciences, catalog number: 556432, Dilution: 1:100
anti-CD81 Rabbit mAb supplier: Cell Signaling Technology, catalog number: 10037S, Dilution: 1:100
anti-HSP60 Rabbit mAb supplier: Cell Signaling Technology, catalog number: 12165s, Dilution: 1:100
anti-CD63 Mouse mAb supplier: Thermo Fisher Scientific, catalog number: 10628D, Dilution: 1:100
anti-CD68 Rat mAb supplier: Invitrogen, catalog number: 14-0681-82, Dilution: 1:100
Goat anti-rat secondary antibody supplier: Thermo Fisher Scientific, catalog number: A-11007, Dilution 1:200
Goat anti-Rabbit IgG Alexa Fluor 488 supplier: Thermo Fisher Scientific, catalog number: A-11034, Dilution 1:200
Goat anti-Mouse IgG Alexa Fluor 488 supplier: Thermo Fisher Scientific, catalog number: A-11029, Dilution 1:200
Goat anti-Rabbit IgG Alexa Fluor 594 supplier: Thermo Fisher Scientific, catalog number: A-11037, Dilution 1:200
Goat anti-Mouse IgG Alexa Fluor 594 supplier: Thermo Fisher Scientific, catalog number: A-11032, Dilution 1:200
For Flowcytometry:
FITC anti-mouse CD45 Antibody supplier: Biolegend, catalog number: 14-0681-82, Dilution: 0.25 μg per million cells in 200 μl volume
PE Anti-Human/Mouse CD11b (M1/70) Antibody supplier: Tonbo Bioscience, catalog number: 50-0112, Dilution: 0.25 μg per million cells in 200 μl volume
F4/80 Monoclonal Antibody (BM8), APC supplier: Invitrogen, catalog number: 17-4801-82, Dilution: 2 μg per million cells in 200 μl volume

Validation

Only commercial and validated antibodies have been used. The validation of each primary antibody for the species and application can be found on the following manufacturer websites.
anti-Rab7 Rabbit mAb https://www.cellsignal.com/products/primary-antibodies/rab7-d95f2-xp-rabbit-mab/9367
anti-Alix Mouse mAb: https://www.cellsignal.com/products/primary-antibodies/alix-3a9-mouse-mab/2171
anti-CD81 Rabbit mAb https://www.cellsignal.com/products/primary-antibodies/cd81-d5o2q-rabbit-mab/10037
anti-CD81 Rabbit mAb https://www.cellsignal.com/products/primary-antibodies/cd81-d3n2d-rabbit-mab/56039
anti-Tom20 Rabbit mAb https://www.cellsignal.com/products/primary-antibodies/tom20-d8t4n-rabbit-mab/42406
anti-Rab4 Rabbit pAb https://www.cellsignal.com/products/primary-antibodies/rab4-antibody/2167
anti-Rab5 Rabbit mAb https://www.cellsignal.com/products/primary-antibodies/rab5-c8b1-rabbit-mab/3547
anti-Rab9A Rabbit mAb https://www.cellsignal.com/products/primary-antibodies/rab9a-d52g8-xp-rabbit-mab/5118

March 2021

anti-Rab11 Rabbit mAb https://www.cellsignal.com/products/primary-antibodies/rab11-d4f5-xp-rabbit-mab/5589
anti-LC3A/B Rabbit pAb https://www.cellsignal.com/products/primary-antibodies/lc3a-b-antibody/4108
anti-Rab27A Rabbit mAb https://www.cellsignal.com/products/primary-antibodies/rab27a-d7z9q-rabbit-mab/69295
anti-Arl8b Rabbit pAb https://www.cellsignal.com/products/primary-antibodies/arl8b-antibody/56085
anti-Atg5 Rabbit mAb https://www.cellsignal.com/products/primary-antibodies/atg5-d5f5u-rabbit-mab/12994
anti-Atg7 Rabbit pAb https://www.cellsignal.com/products/primary-antibodies/atg7-antibody/2631
anti-SQSTM1/p62 Mouse mAb https://www.abcam.com/products/primary-antibodies/sqstm1--p62-antibody-2c11-bsa-and-azide-free-ab56416.html
anti-Tsg101 Mouse mAb https://www.abcam.com/products/primary-antibodies/tsg101-antibody-4a10-bsa-and-azide-free-ab83.html
anti-Calreticulin Rabbit mAb https://www.abcam.com/products/primary-antibodies/calreticulin-antibody-epr3924-er-marker-ab92516.html
anti-Tim23 Rabbit pAb https://www.ptglab.com/products/TIMM23-Antibody-11123-1-AP.htm
anti-CD63 Rabbit pAb https://www.thermofisher.com/antibody/product/CD63-Antibody-Polyclonal/PA5-92370
anti-dendra2 Rabbit pAb https://www.origene.com/catalog/antibodies/tag-antibodies/ta150090/anti-dendra2-rabbit-polyclonal-antibody
anti-GAPDH Mouse mAb https://www.genetex.com/Product/Detail/GAPDH-antibody-GT239/GTX627408
Anti-Ubiquitin (P4D1) Mouse mAb https://www.scbt.com/p/ubiquitin-antibody-p4d1?gclid=Cj0KCQjwilOmBhDjARIsAP6YhSXtB2DykiEspQMGtlj-sRU5j-4pIPp6t2lGrQsAJg7GUkZJKZ9XvcIaArcaEALw_wcB
Anti-MTCO1 Mouse mAb https://www.thermofisher.com/antibody/product/MTCO1-Antibody-clone-1D6E1A8-Monoclonal/459600
Anti-MnSOD Rabbit pAb https://www.emdmillipore.com/US/en/product/Anti-Mn-SOD-Antibody,MM_NF-06-984
anti-Cytochrome c Mouse 6H2.B4 mAb https://www.bdbiosciences.com/en-us/products/reagents/microscopy-imaging-reagents/immunofluorescence-reagents/purified-mouse-anti-cytochrome-c.556432
anti-HSP60 Rabbit mAb https://www.cellsignal.com/products/primary-antibodies/hsp60-d6f1-xp-rabbit-mab/12165
anti-CD63 Mouse mAb https://www.thermofisher.com/antibody/product/CD63-Antibody-clone-Ts63-Monoclonal/10628D
anti-CD68 Rat mAb https://www.thermofisher.com/antibody/product/CD68-Antibody-clone-FA-11-Monoclonal/14-0681-82
FITC anti-mouse CD45 Antibody https://www.biolegend.com/en-us/products/fitc-anti-mouse-cd45-antibody-9796
PE Anti-Human/Mouse CD11b (M1/70) Antibody https://cytekbio.com/products/pe-anti-human-mouse-cd11b-m1-70?variant=40581210374180
F4/80 Monoclonal Antibody (BM8), APC https://www.thermofisher.com/antibody/product/F4-80-Antibody-clone-BM8-Monoclonal/17-4801-82
Goat anti-rat secondary antibody https://www.thermofisher.com/antibody/product/Goat-anti-Rat-IgG-H-L-Cross-Adsorbed-Secondary-Antibody-Polyclonal/A-11007
Goat anti-mouse HRP secondary antibody: https://www.thermofisher.com/antibody/product/Goat-anti-Mouse-IgG-H-L-Secondary-Antibody-Polyclonal/31430
Goat-anti-rabbit HRP secondary antibody: https://www.thermofisher.com/antibody/product/Goat-anti-Rabbit-IgG-H-L-Secondary-Antibody-Polyclonal/31460
Goat anti-Rabbit IgG Alexa Fluor 488 https://www.thermofisher.com/antibody/product/Goat-anti-Rabbit-IgG-H-L-Highly-Cross-Adsorbed-Secondary-Antibody-Polyclonal/A-11034
Goat anti-Mouse IgG Alexa Fluor 488 https://www.thermofisher.com/antibody/product/Goat-anti-Mouse-IgG-H-L-Highly-Cross-Adsorbed-Secondary-Antibody-Polyclonal/A-11029
Goat anti-Rabbit IgG Alexa Fluor 594 https://www.thermofisher.com/antibody/product/Goat-anti-Rabbit-IgG-H-L-Highly-Cross-Adsorbed-Secondary-Antibody-Polyclonal/A-11037
Goat anti-Mouse IgG Alexa Fluor 594 https://www.thermofisher.com/antibody/product/Goat-anti-Mouse-IgG-H-L-Highly-Cross-Adsorbed-Secondary-Antibody-Polyclonal/A-11032

Eukaryotic cell lines

Policy information about cell lines and Sex and Gender in Research

Cell line source(s)

WT and Atg5-/- Mouse embryonic fibroblast (MEF) were obtained from Dr. Noboru Mizushima (The University of Tokyo, Japan). WT and Rab7-/- MEFs were generously provided by Dr. Edinger (UC Irvine). Raw 264.7 macrophages were initially obtained from ATCC and provided by Dr. Anthonio De Maio (UC San Diego) for this study.

Authentication

Generation and authentication of WT and Rab7-/- MEFs were described by Dr. Aimee Edinger and colleagues (see Roy et al. Autophagy (2013) 9(7): 1009-23. Rab7 deletion has also been confirmed as a part of this study by Western blotting for Rab7 protein levels. The WT and Rab5-/- were generated and authenticated by Kuma et al. Nature 432:1032-36, 2004. Loss of Atg5 and the resulting deficiency in autophagosome formation have been verified by Western blotting experiments.

Mycoplasma contamination

Cells lines were subjected to examination of mycoplasma contamination by using the LookOut Mycoplasma PCR Detection Kit purchased from Sigma (MP0035-1KT). All cell lines used for experiments tested negative for mycoplasma.

Commonly misidentified lines
(See ICLAC register)

None of the cell lines used in this work were listed as "Misidentified Cell Line" in the ICLAC database

Animals and other research organisms

Policy information about studies involving animals; ARRIVE guidelines recommended for reporting animal research, and Sex and Gender in Research

Laboratory animals

Rab7f/f mice were crossed with cardiac specific Myh6-MerCreMer (MCM) (Jackson Laboratory Stock #005650) to generate cardiomyocyte-specific Rab7 knockout mice. Cre negative littermates were used as controls. To selectively delete Rab7 in myocytes, male and female mice 8-10 weeks of age were injected (i.p.) with 40 mg/kg tamoxifen (Sigma-Aldrich, T5648) for five consecutive days. Control mice were also injected with tamoxifen. Mice with cardiac specific expression of mito-Dendra2 were generated by

	breeding female PhAM-floxed (Jackson Laboratory, Stock #018385) and male Myh6-Cre or Myh6-MerCreMer old mice. These mice were used for experiments at 8-10 weeks of age. 8-12 weeks old Lamp-2 knockout male mice were used in this study. Young (4-month-old) and aged (24-month-old) male C57BL/6 mice were obtained from the National Institute of Health Institute of Aging colony (Charles River). Mice were housed in a 12h light/dark environment at a temperature of 20.5-21.5°C and with 30-60% humidity.
Wild animals	This study did not involve wild animals.
Reporting on sex	Both male and female mice were used in this study and sex was not considered in the study design since we did not observe a difference in EV secretion between males and females. The only exceptions are experiments involving LAMP2-/- and aged mice. The Danon disease due to LAMP2 mutations is characterized by an X-linked dominant inheritance pattern and males are more severely affected than females. Due to limited availability of aged mice in the NIA aging colony, only male mice were obtained and used for experiments.
Field-collected samples	This study did not involve samples collected from the field.
Ethics oversight	All animal experiments were performed following the Guidelines of National Institutes of Health on the Use of Laboratory Animals and approved by the Institutional Animal Care and Use Committee at the University of California, San Diego.

Note that full information on the approval of the study protocol must also be provided in the manuscript.

Flow Cytometry

Plots

Confirm that:

☒ The axis labels state the marker and fluorochrome used (e.g. CD4-FITC).

☒ The axis scales are clearly visible. Include numbers along axes only for bottom left plot of group (a 'group' is an analysis of identical markers).

☒ All plots are contour plots with outliers or pseudocolor plots.

☒ A numerical value for number of cells or percentage (with statistics) is provided.

Methodology

Sample preparation	Adult mouse heart was excised, cannulated, and perfused with Liberase DH (26U/ml, Roche) in buffer containing 110 mM NaCl, 4.7 mM KCl, 0.6mM NaHPO4, 0.6 mM KH2PO4, 1.25 mM MgSO4, 10 mM KHCO3, 12 mM NaHCO3, 5.5 mM glucose, 30mM Taurine and 10 mM HEPES (pH 7.4). After digestion, ventricular tissue was gently teased into small pieces to dissociate loose cells. After about 30 min of sedimentation, the supernatant was centrifuged, and the cell pellet resuspended in staining buffer (BioLegend). Cells were seeded in 96-well plate, incubated with antibodies against CD45 (BioLegend, clone I3/2.3), CD11b (Tonbo Bioscience, clone M1/70) and F4/80 (Invitrogen, clone BM8), and then analyzed using a Guava benchtop mini-flow cytometer (EMD Millipore).
Instrument	Samples were analyzed using a Guava benchtop mini-flow cytometer (EMD Millipore).
Software	Data were qualified using FlowJo software (Version 10.8.1)
Cell population abundance	n/a - experiments did not involve post-sort fractions
Gating strategy	Gating strategy used to identify cardiac macrophage subsets has been shown in Supplementary Fig. 6f

☒ Tick this box to confirm that a figure exemplifying the gating strategy is provided in the Supplementary Information.
